# AIEgen orthopalladated hybrid polymers for efficient inactivation of the total coliforms in urban wastewater

**DOI:** 10.1038/s41598-023-41315-x

**Published:** 2023-09-22

**Authors:** Lucia Sessa, Rosita Diana, Francesco Silvio Gentile, Fabio Mazzaglia, Barbara Panunzi

**Affiliations:** 1 Department of Pharmacy, University of Salerno, Via Giovanni Paolo II, 132, 84084 Fisciano, SA Italy; 2 Department of Agricultural Sciences, University of Naples Federico II, Portici, NA Italy; 3 Department of Chemical Sciences, University of Napoli Federico II, Strada Comunale Cinthia, 26, 80126 Napoli, Italy; 4 C.R.A. S.R.L., Calle Giovanni Legrenzi, 2, 30171 Venice, VE Italy

**Keywords:** Chemistry, Materials science, Optics and photonics

## Abstract

Monitorable AIE polymers with a bioactive pattern are employed in advanced biomedical applications such as functional coatings, theranostic probes, and implants. After the global COVID-19 pandemic, interest in developing surfaces with superior antimicrobial, antiproliferative, and antiviral activities dramatically increased. Many formulations for biocide surfaces are based on hybrid organic/inorganic materials. Palladium (II) complexes display relevant activity against common bacteria, even higher when compared to their uncoordinated ligands. This article reports the design and synthesis of two series of orthopalladated polymers obtained by grafting a cyclopalladated fragment on two different *O, N* chelating Schiff base polymers. Different grafting percentages were examined and compared for each organic polymer. The fluorescence emission in the solid state was explored on organic matrixes and grafted polymers. DFT analysis provided a rationale for the role of the coordination core. The antibacterial response of the two series of hybrid polymers was tested against the total coliform group of untreated urban wastewater, revealing excellent inactivation ability.

## Introduction

Demand for responsive polymeric materials is continuously growing. Chromophoric advanced tools focused the attention of scientists and technologists due to their extensive uses in biotechnology and biomedical sciences. Their role as drug-carriers, anti-cancers, antimicrobials, sensors, and markers is well documented^1–6^. Specifically, chromophores exhibiting aggregation-induced emission (AIEgens) are the subject of keen interest^7,8^. AIEgens^9–11^ are luminophores active in the aggregate state and concentrated solutions. Therefore, they are active in aqueous and/or physiological environments where they aggregate.

The combination of AIE activity and stimuli responsiveness in a single platform provides an effective strategy for developing novel polymeric tools for modern bio-applications. Furthermore, thanks to the state-of-the-art bioimaging technologies, macro or nanoformulation of monitorable bioactive AIE-polymers provide theranostic probes for therapy and diagnostics^12,13^.

Antimicrobial resistance is one of the major challenges in human history. Macromolecules have been efficiently and extensively employed in the biomedical field as antimicrobial probes^14,15^. Due to the current global COVID-19 pandemic, interest in developing novel polymeric scaffolds with superior antibacterial and antiviral activities dramatically increased^16^. Antimicrobial polymers are desirable as surface coatings preventing viable bacteria adhesion^17^, for tissue engineering applications^18^, and as antibiofilm in healthcare and food packaging^19^. Fully organic antimicrobial polymers are abundant and well-documented^20,21^. In addition, many formulations for antimicrobial surfaces are based on the hybrid combination of an inorganic antimicrobial moiety with an organic one^22^. Many pharmaceutical agents in current clinical use containing metals^23^, and polymer-metallic composites are widely employed in the smart-packaging industry^24^ for the production of active biofilms^25^ and for the synthesis of nanosized theranostic instruments with high activity and broad response spectrum^25,26^. From the rational design of the structure–activity relationship, novel bio-responsive hybrid materials have been synthesized where the pivotal role of the metal cation is fully recognized^27–32^.

Widely used in organic synthesis^33,34^, Schiff bases are known as biologically performant molecules^35,36^. Very recently, the antimicrobial properties of a series of molecules containing salicyl-aldehyde groups were examined from both the experimental and the theoretical points of view^37,38^. The manageable Schiff base synthesis is the starting point to produce organic polymers able to chelate a suitable metal central cation. In many of the resulting hybrid materials, the biological/pharmacophore effects can be improved, including antifungal, antimicrobial, antiproliferative, anti-inflammatory, and antiviral activity^21,27,33,39^.

Palladium organometallic complexes are widely employed in bioinorganic chemistry^40,41^. Organopalladium tools are good inactivators of the world’s best-characterized, ubiquitous, and adaptable organism, *Escherichia coli*^42,43^. *Escherichia coli* is the most representative microbial species of the faecal coliform group, in turn, part of the larger family of total coliforms, present in the digestive tracts of animals, in wastes, in plants, and in soil material. Therefore, total coliform counts give a general indication of the sanitary condition of water, aqueous environments, and humid surfaces. Even better, testing for coliform bacteria can be a reasonable indication of whether other pathogenic bacteria are present^44^.

In previous works, we systematically investigated orthopalladated push–pull Schiff bases as stable chromophores and/or bioactive molecules^45–47^. The antagonist response of some low-weight palladium complexes to *Escherichia coli* bacteria gave interesting results that prompted us to expand the study towards polymeric systems.

**Figure 1:**
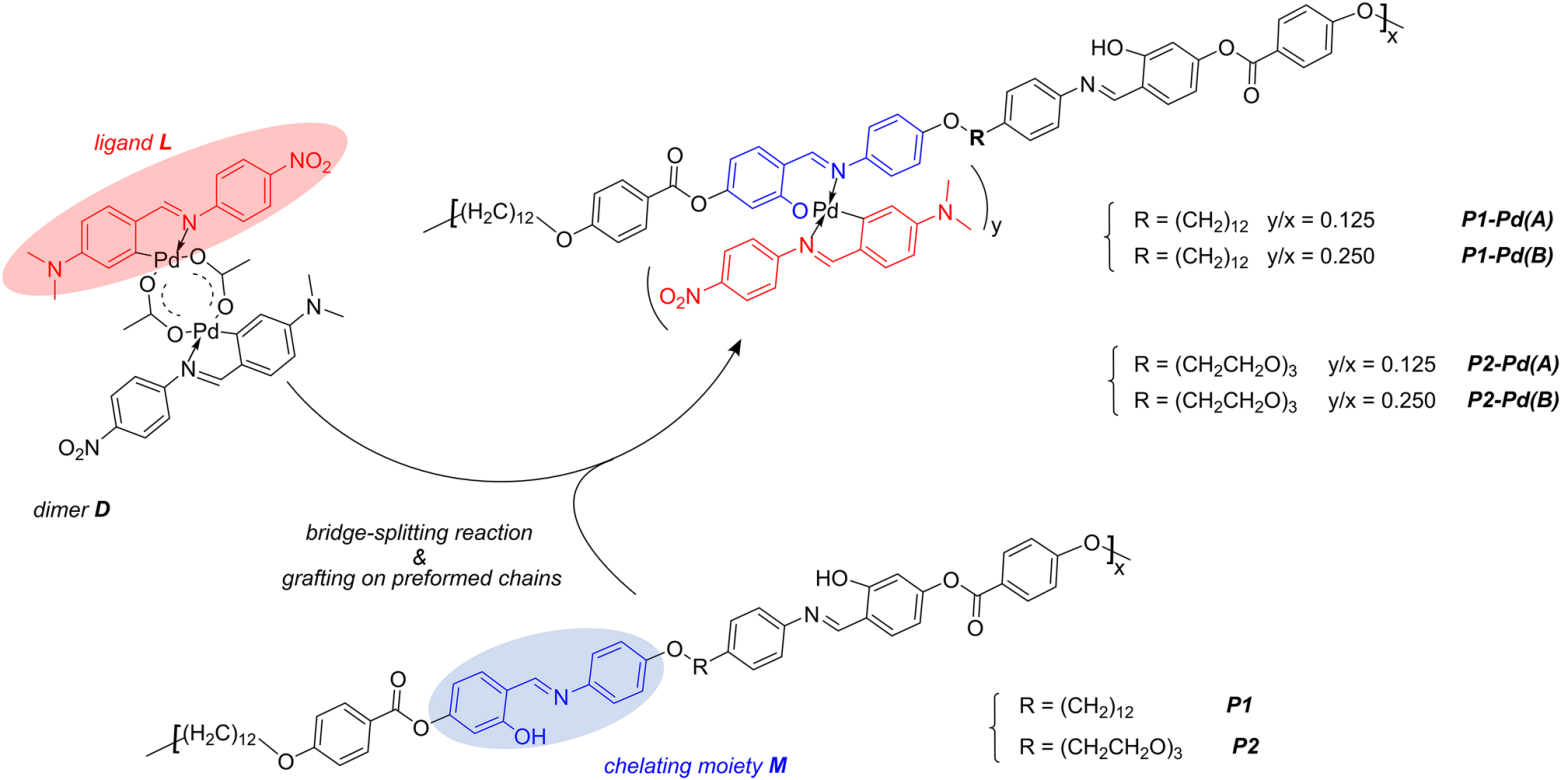
Synthetic route to the hybrid polymers.

This article reports the design and synthesis of a series of hybrid metallopolymers obtained by grafting the orthopalladated fragment Pd-L (see Fig. 1) on the *O*, *N* chelating sites of two Schiff base polymers (P1 and P2, see Fig. 1) with a different flexible spacer. The two organic polymers are AIE active materials with a solid-state light-yellow emission. They can be moulded and formed in thin layers and display by-contact antimicrobial activity against the total coliform group. To preserve emission and solubility, we grafted the orthopalladated fragment one site out of sixteen (samples named A) or one site out of eight (samples named B) chelating units, obtaining two hybrid series (see Fig. 1). The grafted polymers still display a solid-state emission gradually red-shifted depending on the organometallic fragment content. A comprehensive exploration of chemical-physical properties, optical behaviour, and antimicrobial assays was performed. Specifically, a good-to-excellent activity was recorded against the total coliform group, which significantly increased from the organic to the hybrid polymers. By correlating experimental data and theoretical DFT investigation, we could argue the pivotal role of the coordination core as an active electron bridge across the ligand system.

## Experimental section

### Materials

Commercially available starting products were supplied by Sigma Aldrich. Compound D was obtained as described in Refs.^48,49^.

^1^H NMR spectra were recorded in 1,1,2,2-tetrachlorethane-d_2_ with a Bruker Advance II 400MHz apparatus by Bruker Corporation, Billerica, MA, USA. Optical observations were performed using a Zeiss Axioscop polarising microscope by Carl Zeiss, Oberkochen, Germany, equipped with an FP90 Mettler microfurnace by Mettler-Toledo International INC MTD, Columbus, OH, USA. The decomposition temperatures (measured as 5 wt% weight loss) and phase transition temperatures and enthalpies were measured under nitrogen flow 50 mL/min, using a DSC Mettler Toledo DSC3+ at a scanning rate 10 °C min^−1^. TGA experiments were performed by a thermobalance Perkin Elmer Pyris Diamond TGA under airflow 25 mL/min, at a scanning rate 20 °C min^−1^. Absorption and UV–Visible emission spectra were recorded using JASCO F-530 and FP-750 spectrometers by JASCO Inc., Easton, MD, USA, at a scanning rate of 200 nm min^−1^, and on a spectrofluorometer Jasco FP-750 by JASCO Inc., Easton, MD, USA (excitation wavelengths set at the absorption maxima of the samples) at scanning rate 125 nm min^−1^. Thin films of the samples were prepared using an SCS P6700 spin coater operating at 600 rpm for 1 min (I step) and at 3000 rpm for 30 s (II step). Photoluminescence quantum efficiency values were recorded on quartz slides using a Fluorolog 3 spectrofluorometer by Horiba Jobin Instruments, SA, within an integrating sphere provided by an optical fibre connection. Morphological characterization was performed by a Scanning Electron Microscope (SEM) Leo 1530 Gemini by Zeiss on the spun films. The operating voltage was 6 kV for all the measurements performed. The polymers P1 and P2 intrinsic viscosity (η) at 130.0 °C in o-dichlorobenzene was measured with an Ubbelohde viscometer. Vapour-pressure osmometry (Knauer apparatus) of o-dichlorobenzene solutions at 130 °C was utilized to estimate the average molecular weights (MW). X-ray powder diffraction data were collected in reflection geometry using the Empyrean (PANalytical) diffractometric apparatus using Nifiltered Cu Kα radiation (λ = 0.15418 nm).

### Organic polymers

Polymers P1 and P2 were obtained following a procedure described by us^50^. Polymerization was performed in solution starting from two different dianiline monomers (with different flexible spacers for P1 and P2) and the same dialdehyde monomer. Formulas of the precursors are reported in Fig. S1. As an example, in the synthesis of P1 a total of 4.397 g of the dialdehyde monomer bis(4-formyl-3-hydroxyphenyl) 4,4′-(dodecane-1,12-diylbis(oxy))dibenzoate was dissolved in 40 mL of boiling o-dichlorobenzene and 2.477 g of the dianiline monomer 4,4′-(dodecane-1,12-diylbis(oxy))dianiline was added under a nitrogen atmosphere. The reaction mixture was kept under vigorous stirring at boiling temperature, then poured into boiling n-hexane. The precipitated polymer was washed twice in n-hexane and oven-dried at 100 °C. Yields are virtually quantitative. The reaction time for the polycondensation was modulated to obtain medium molecular weight, suitable viscosity, and good adhesion properties. Specifically, we kept the reaction for 20′ in boiling o-dichlorobenzene. Intrinsic viscosity measurement in o-dichlorobenzene at 130 °C provided values of 2.50 dL/g and 1.65 dL/g for P1 and P2, respectively. The ^1^H-NMR spectrum of P1 and P2 (Figs. S2, S3) are consistent with the expected formula. Terminal groups are barely detectable at 11.20 ppm (OH proton) and 9.89 ppm (CHO proton) for both P1 and P2 spectra. The ratio between the areas of the diagnostic peaks of the polymers and the peaks due to the terminal groups at least ten monomer units in the polymeric chain. Vapour-pressure osmometry by a Knauer apparatus was used to measure the average molecular weights (MW) of P1 and P2 in o-dichlorobenzene at 130 °C. In both cases, we obtained a value ranging from 12 × 10^3^ to 13 × 10^3^. A detailed analysis of the thermal behaviour of the samples was performed in our previous article. The samples employed in this article replicate the expected thermal behaviour (see “Antibacterial activity” section). X-ray diffraction pattern recorded on P1 and P2 samples confirm a small amount of crystallinity^50^. In both cases, the solid samples melt to a mesophase (anisotropic nematic liquid) observable in crossed polarizers optical microscopy as a schlieren texture.

### Synthesis of the grafted polymers

The grafted polymers were obtained by bridge-splitting reaction of the complex D on a preformed chain of P1 or P2 polymer (see Fig. 1). The grafting reaction was carried out by the same procedure. As an example, we describe the reaction to obtain P1-Pd(B). To 1.030 g of P1 (molecular unit: C_64_H_74_N_2_O_10_, 1030.53) dissolved in 15 mL of boiling 1,1,2,2-tetrachlorethane (TCE) 0.108 g of D (0.125 mmol) dissolved in 3 mL of hot TCE. To the reaction solution, 200 mg of K_2_CO_3_ was added, and the boiling mixture was kept under stirring for 2 h. The crude product was filtered and precipitated by pouring methanol at room temperature. The compound was washed in methanol twice and dried at 80 °C overnight under a vacuum. Yield is virtually quantitative. The ^1^H-NMR spectra of all grafted polymers were reported in Supplementary Materials (Figs. S4–S7). Referring to the NMR pattern, the insertion of the Pd-L fragment is revealed by some diagnostic signals, although scarcely intense due to the low grafting degree. Compared to NMR spectra of the unbonded polymers, NMR spectra of all the hybrid polymers show two very small signals in the region from 5 to 6.5 ppm, due respectively to the proton in ortho and in para to the C-Pd bond, and a singlet is in the region from 2.5 to 3 ppm due to NMe_2_ protons. For comparison, the spectrum of compound D showing the same unique pattern can be seen in Fig. S8.

### Molecular modelling

Ab-initio calculations were performed in the Density Functional Theory (DFT) approximation using the Linear Combination of Atomic Orbitals (LCAO) formalism. Then, the molecular orbitals were expanded in Gaussian Atomic Orbital (GAO) functions centred in the nuclei.

HOMO–LUMO analysis is obtained from the ground and first excited state calculations; in particular, the latter is achieved by adopting the Time-Dependent Density Functional Theory (TDDFT) formalism.

The software adopted is ORCA 5.0.3^51^. A B3LYP hybrid functional^52^, with a 20% fraction of the exact Hartree Fock exchange, was used as previously described^53,54^. The dispersion interactions are evaluated by adopting the D3 Grimme empirical correction Double-z (for the light atoms) and Large Triple-z (for the metal atom) basis set was used. In this case, the def-SVP and def-TZVP basis sets of the Karlsruhe group were adopted^55^. This basis set was previously tested by us^53,54,56^. Therefore, they can be considered a good choice in terms of computational performance and accuracy of the results, providing a reliable comparison with the experimental data without renouncing a smart calculation.

### In vitro antibacterial activity methods

The reference sample for colony development is urban wastewater collected on the Tyrrhenian side of Italy. The sample contains a collection of different bacteria, viruses, and parasites. As expected, microbiological analysis evidenced an abundance of *Escherichia, Enterobacter*, *Citrobacter*, and *Klebsiella*.

The standard 7010 C APAT IRSA-CNR method was followed for the collection, storage, and preparation of the samples and to evaluate the bacterial colonies. The wastewater was diluted 1: 1000 times to allow the optimal number of colonies to be read. A 100 mL aliquot was taken and placed in membrane filtration for each test. The wastewater sample was treated by filtering a water content of 100 mL on a nitrocellulose membrane with a porosity of 0.45 µm. The filtration system consists of a ramp with supports and containers (filter funnels) made of stainless steel, using an electrically operated vacuum pump or a water pump as a suction system. All operations are performed in sterile conditions, the supports and containers being sterilized in an autoclave or sterile disposable. Bacteria larger than the filter’s pores remain trapped on the surface of the filter itself. After filtration, each membrane is placed in a plate containing a specific culture medium that allows the growth of the bacteria.

In our case, the medium used for total coliforms was chromogenic coliform agar. Each polymer sample was dispersed in chloroform at a concentration equal to 1 mg/mL, i.e., 0.1% W/V. Each emulsion of the polymer samples undergoes sonication for 30′. On each 6 mm Petri dish agarised with m-Endo agar (Less), 5 mg of the polymer was homogeneously dispersed on the surface. The dispersion was carried out using a polymer suspension deposited on the agar, then evaporated under aseptic conditions in a biological hood.

The plates prepared with known concentrations of the tested complex were incubated at 37 °C for 24 h for both the investigated microorganisms. After incubation, the results were read by comparing the blank or without complex plates and those with the complex, observing a significant decrease in the number of colonies.

## Results

### Chemistry of the grafted polymers

Developing novel optical and biological-responsive tools meets the expectations of technological and scientific demands. Biologically performant macro or nano-sized AIE polymers^11,57–60^ play a key role among the modern monitorable theranostic tools^61,62^. Hybrid polymers obtained by grafting metalloorganic fragments on preformed chelating polymers combine the easy and tuneable synthesis with the specific features brought by the coordination fraction.

The grafting process synthetic strategy provides an easy-not-trivial alternative to the common practice of doping active molecules in a polymeric host matrix. Using preformed polymeric chains with suitable chelating sites leads to well-defined MW material where the active units are chemically bonded. Moreover, the grafting percentage can be turned to optimize desirable properties. Metals used as bridges along a conjugated organic system are expected to induce a more efficient electron π-conjugation path with significant spectroscopic and even biological differences between free ligands and metal complexes^63–65^.

The Pd–C organometallic bound was exploited to firmly graft non-symmetric coordination moieties on preformed polymeric chains in different percentages^66,67^. In the first synthetic step, the Schiff base L in Fig. 1 undergoes orthopalladation resulting in the aceto-bridged dinuclear species D. By the bridge-splitting reaction of D directly on the *O,N* chelating sites of P1 or P2 substrates, we grafted the Pd-L complex moiety on the polymeric skeletons (see Fig. 1), in different percentages.

As for the aceto-bridged dinuclear species D, the coordination moiety proved to have an excellent antimicrobial potential^48^. The ligand L has a π-electron conjugated system, dying properties, and proven antimicrobial activity, which increases in some related complexes^68,69^. Locked in the orthopalladated ring, the iminic bond of L benefits from stabilization. At the same time, the reaction of the orthopalladated Pd-L fragments with the chelating M sites of P1 and P2 produces a second stable, rigid cycle. The big differences between organic and hybrid polymers must be ascribed to the electron charge transfer involving the whole coordination core, as discussed in “Theoretical analysis” section^70^.

The organic polymers P1 and P2 are medium MW Schiff base polymers obtained by polycondensation reaction of a salicylic di-aldehyde and a di-aniline. Two equivalent *O*, *N* chelating sites for each monomer unit derive from the polycondensation reaction. The flexible spacers guarantee solubility and processability. Two different di-aniline were employed with different flexible chains. The most articulated triethylene glycol (TEG) spacer used in the synthesis of P2 ensues greater (about 30%) solubility compared to P1. Hence the indisputable advantage of better processability. Both polymers were expected to be emissive and pathogen antagonists^34,36,37,71^. Moreover, P2 was expected to be a better pathogen antagonist than P1. PEG (polyethylene glycol) based polymers exhibit a broad spectrum of antimicrobial activities and are employed as long-lasting antimicrobial surface coatings able to reduce the adhesion of bacteria^72–76^.

By tuning the grafting percentage of the organometallic fragment, we did search for the best balance between spectroscopic and biological properties. Specifically, we set the Pd-L moiety content as 0.25 mol ratio (Pd-L/Organic polymer) in type (A) samples and 0.125 mol ratio (Pd-L/organic polymer) in type (B) samples. Therefore, as two chelating sites are available per monomer unit both in P1 and P2, we grafted one site in sixteen in the case of the series named (A) and one site in eight in the series named (B) (see Fig. 1). As a percentage, type (A) samples were obtained using dinuclear complex D, about 5% w/w of the total, and type (B) samples using dinuclear complex D, about 10% w/w of the total. By not exceeding 10% D, we planned to achieve good antimicrobial ability without sacrificing the solubility and the AIE pattern.

### Antibacterial activity

Many scientific studies have focused on the removal of bacteria^77^. Palladium (II) complexes from Schiff bases ligands are known for their significant activity against Staphylococcus aureus and Gram-negative bacteria, even higher compared to their free respective ligands. The group of total coliforms are rod-shaped, Gram-negative, mainly aerobic, and non-sporing bacteria. The group is a collection of Gram- negative bacteria, including *Citrobacter*, *Enterobacter*, *Hafnia*, *Klebsiella*, some species of *Serratia*, and *Escherichia coli*. They are considered classic indicators of contamination to evaluate the hygienic conditions of home and work environments and food and water.

The total coliform test provided us with extensive information on the inhibitory ability of our polymers. Moreover, we could use a real sample of urban wastewater without any pre-treatment.

A total coliform inhibition test was performed to investigate the ability of both organic polymers and hybrid polymers to mitigate or prevent bacterial growth. The colony-forming units (CFU) were evaluated at the same concentrations of polymer stock suspension (see “In vitro antibacterial activity methods” section). The CFU abatement results are summarized in Table 1.

**Table 1:** CFU abatement results for organic and hybrid polymers.

Sample	CFU/100 mL	CFU (%)	Inhibition efficiency (%)
Control 1	80,000	100	0
P1	57,000	71	29
P1-Pd(A)	1100	1.4	98
P1-Pd(B)	0	0	100
Control 2	160,000	100	0
P2	80,000	50	50
P2-Pd(A)	75,000	46	54
P2-Pd(B)	35,000	22	78

Standard 7010 C APAT IRSA-CNR method was used, as described in “In vitro antibacterial activity methods” section.

Due to the Schiff base M active moiety (see Fig. 1), unfunctionalized P1 and P2 samples were found to affect the growth of the total coliforms sample. As expected, the presence of a TEG spacer makes the biocide activity of P2 higher (quite double) with respect to P1^63–65,70^.

All the hybrid polymers show over 50% inhibition of CFU, higher than the respective unfunctionalized polymer. By increasing the grafting percentage, the biocidal activity increases for both grafted series, i.e., samples (A) have lower activity than samples (B). Both P1-Pd(A) and P1-Pd(B) reach quite 100% CFU abatement. This is a remarkable result as compared with recent literature data^78^.

**Figure 2:**
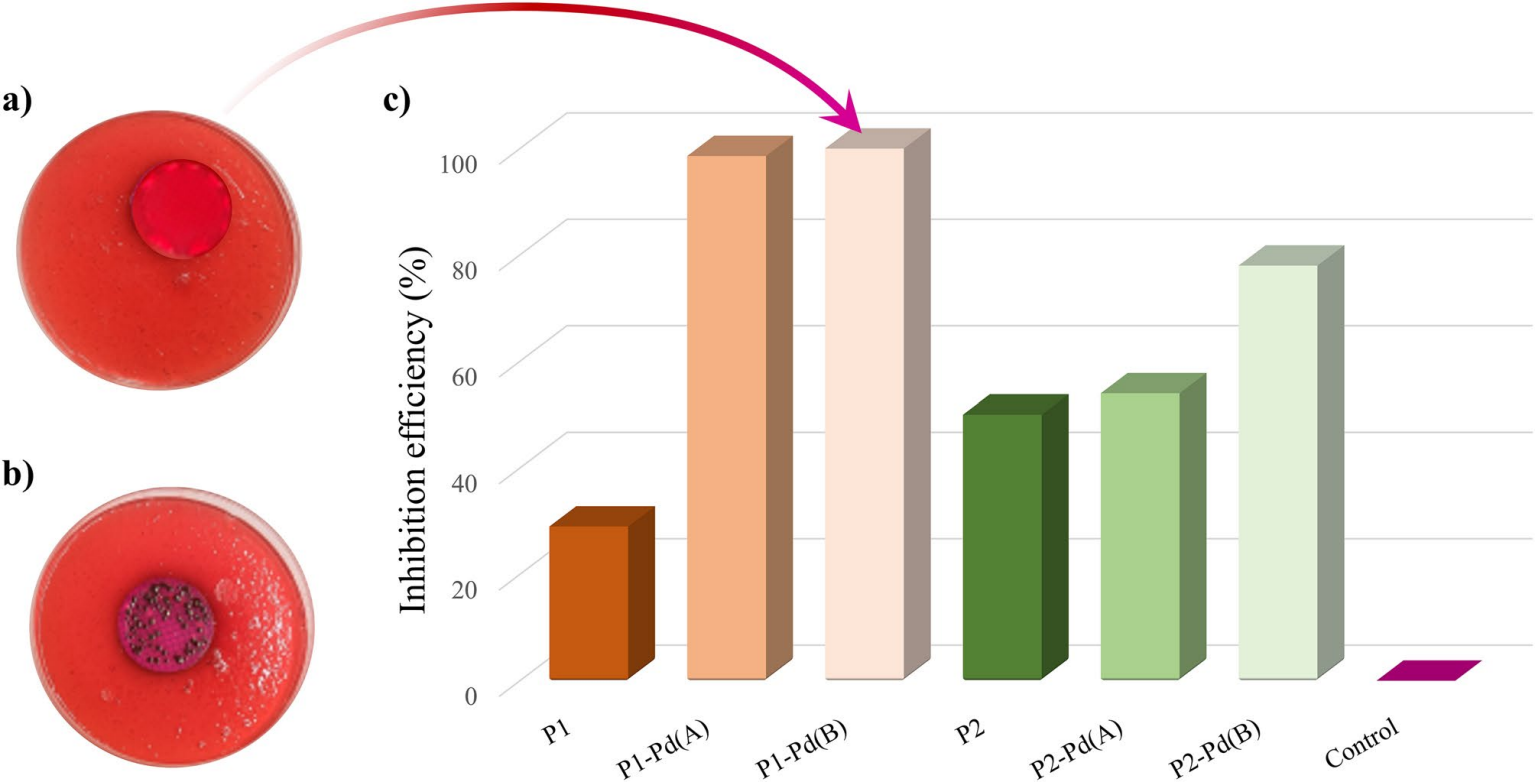
Petri dishes of the wastewater sample for P1-Pd(B) (**a**) and control (**b**). Percentage inhibition efficiency of all the polymers by standard 7010 C APAT IRSA-CNR method (**c**).

Contrary to what was expected, P2-Pd derivatives show antibacterial activity significantly lower than Pd1-Pd derivatives (Table 1). Structure-dependent antimicrobial activities were discussed in significant articles^79–81^. In addition to the surface effects due to the different spacers^82^, we could assume a role of the metal-chain interactions on the biofilm growth. Ethylene glycol (EG)-functionalized palladium (II) complexes are well known^83,84^. In our case, a crosslinked structure through weak interactions of the oxygen atoms of the TEG chains to the tetracoordinate metal centres may occur. As a result, the tetracoordinate core may distort, and the glycolic chains may change configuration with an unforeseen global effect on the biocidal activity^85,86^. The polymers’ inhibition efficiency is summarised in histograms in Fig. 2c. On the left of the same figure, picks of the Petri dishes treated with the wastewater sample are reported in the case of the most active polymeric sample P1-Pd(B) (Fig. 2a) compared with the control (Fig. 2b).

### Phase behaviour, optical response, and stability

The main-chain organic polymers are yellow semicrystalline materials with a nematic mesophase (see thermal behaviour summarised in Table 2). DSC curves of P1 and P2 (Figs. S9, S10) display high melting points of the crystalline fraction (above 220 °C) with a polymorphic behaviour in the solid state (two melting phenomena reported in Table 2, the least intense in brackets). Decomposition occurs with isotropization.

**Table 2:** Thermal behaviour of organic and hybrid polymers.

Sample	Mp/T_g_ (°C)^a^	T_i_ (°C)^b^	T_d_ (°C)^c^	Pd (%)^d^	Pd (%)^e^	Solubility (mg/mL)^f^
P1	(162) 238^g^	320 (with decomposition)	325	–	–	50
P1-Pd(A)	192	Not detectable	310	1.23	1.20	50
P1-Pd(B)	165 (T_g_)	–	318	2.36	2.25	20
P2	190 (220)^g^	310 (with decomposition)	315	–	–	65
P2-Pd(A)	186	Not detectable	312	1.30	1.24	65
P2-Pd(B)	130 (T_g_)	Not detectable	315	2.48	2.44	40

^a^Melting point or glass transition temperature.

^b^Isotropization temperature.

^c^Decomposition temperature.

^d^Calculated palladium content.

^e^Experimental palladium content.

^f^Solubility in 1,1,2,2-TCE at 100 °C.

^g^Two melting points were detected (the weaker signal is in parentheses).

The introduction of the orthopalladated units forcing the ligand sites in a local planar or semi-planar arrangement causes a loss of the structural order both in the solid state and in the mesophase. The grafted polymers with lower palladium content P1-Pd(A) and P2-Pd(A) (see DSC curves in Figs. S9, S10, respectively) are semicrystalline materials as the organic precursors (see Table 2). After melting, both samples retain a nematic mesophase, clearly observable in crossed polarizers optical microscopy as a poorly mobile schlieren texture. The samples with higher palladium content P1-Pd(B) and P2-Pd(B), display different behaviour. P2-Pd(B) is a non-crystalline sample retaining a nematic mesophase observable as a poorly mobile schlieren texture above T_g_ (above 140°C, see DSC curve in Fig. S10). Decomposition occurs before isotropization. P1-Pd(B) is an amorphous material fluid and isotropic above T_g_ (above 170 °C, see DSC curve in Fig. S9), missing the mesophasic behaviour above T_g_. This material can be deposed as an orange film that is highly transparent (see Fig. 2), a desirable requirement for some coating applications^17,72^. SEM analysis confirmed the homogeneous non-structured nature of the film.

All the polymeric samples show remarkable thermal stability with decomposition above 300 °C. By TGA analysis, we also obtained the Pd (0) content of the metallated polymers. Fixed the percentage of dimer compound D at 5% and 10% (and consequently the percentage of Pd-L fragment), the expected Pd content varies between just over 1% and just over 2% for type A and type B samples, respectively. As can be detected from Table 2, the grafted polymers show quite nominal palladium content proving the grafting route an efficient synthetic way to obtain hybrid polymers.

Organic polymers and grafted polymers in natural light are from yellow to orange materials (see Fig. 2). The intriguing spectroscopic pattern of P1 and P2 was never explored before^50^. Here we checked their absorption/emission properties, highlighting their potential as fluorophores. Specifically, we found that P1 and P2 polymers are poorly emissive in a diluted solution. Conversely, they act in the AIE mode displaying yellow emission in the aggregate phase. Absorbance and emission maxima measured on spin-coated thin films (sample photographed in Fig. S11), RGB emission colour space, Stokes shift, and photoluminescence quantum yields (PLQYs) for both organic and hybrid polymers are summarised in Table 3.

**Table 3:** Spectroscopic behaviour of organic and hybrid polymers.

Sample	λ_ab_ film (nm)^a^	λ_em_ film (nm)^b^	RGB^c^	Stokes shift (nm)^d^	PLQY%^e^
P1	352	518	156,184,63	166	30 ± 0.2
P1-Pd(A)	364	547	204,217,50	183	24 ± 0.2
P1-Pd(B)	357	562	159,103,72	205	2.0 ± 0.2
P2	369	521	166,216,105	152	19 ± 0.2
P2-Pd(A)	351	540	217,244,101	189	14 ± 0.2
P2-Pd(B)	347	548	196,185,97	201	2.5 ± 0.2

^a^Wavelength of UV–Visible absorbance maxima on spin-coated thin films.

^b^Wavelength of emission maxima on spin-coated thin films (excited to absorption maximum wavelength).

^c^RGB emission colour space.

^d^Stokes shift (from emission to absorption).

^e^PL quantum yield measured on thin films.

**Figure 3:**
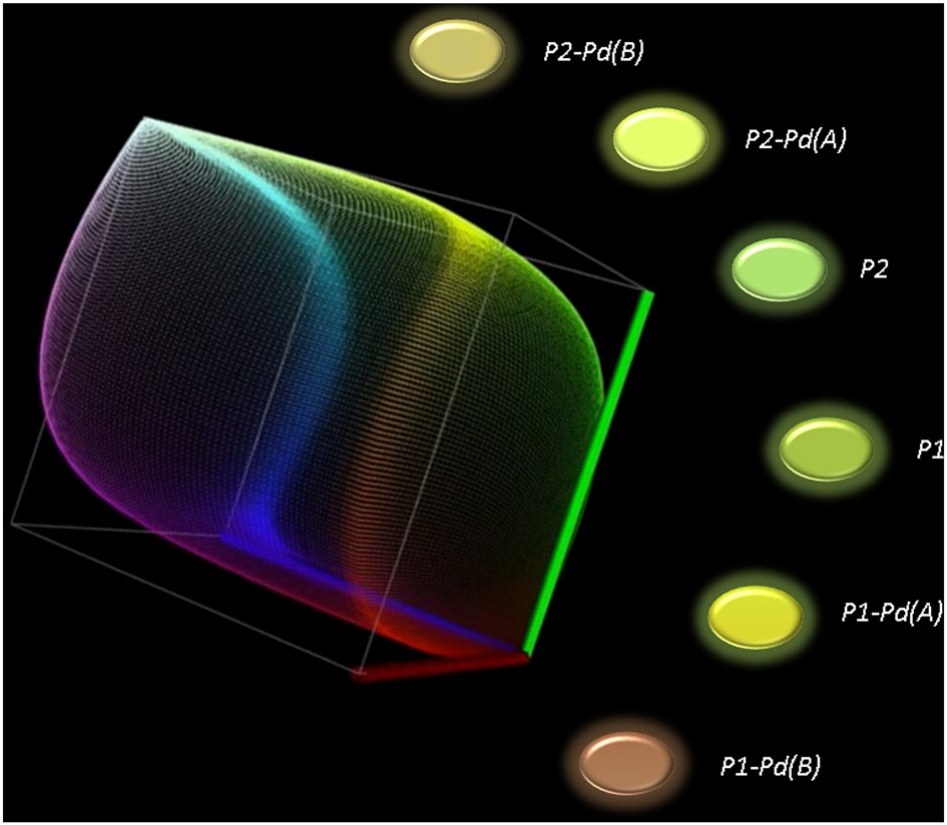
Eye-catcher of the polymers’ emission under a UV–Visible lamp at 365 nm. The ovals were cut out from the real thin film samples and placed from the appropriate side of the tridimensional RGB colour space diagram (red-green-yellow edge).

As expected, Refs.^77,87,88^ the non-emissive Pd-L grafted moiety causes a decrease in the emission performance. Specifically, by increasing Pd-L content from type (A) samples to type (B) samples, the emission band broadens and undergoes a redshift (Figs. S12, S13). At the same time, PLQYs drop. Therefore, P1 and P2 show medium-to-good PLQYs. Type (A) samples still retain medium PLQYs. In addition, Stokes shifts are large (183 nm and 189 nm, respectively) as desirable to minimize emissions-absorption overlap^89,90^. In type (B) samples PLQYs lower to about 2%. Samples with higher palladium content than type (B) were not examined due to the non-emissive pattern and poor solubility (see solubility in Table 2). Interestingly, the yellow/orange fluorescence emission of organic and hybrid polymers (compared in Fig. 3) can be naked eye observed by using the standard 365 nm excitation wavelength of a commercial UV lamp.

The spin-coated films of all grafted samples show strong adhesion to the glass support and resist common organic solvent redissolving and mechanical scratching.

A systematic analysis of the stability in ordinary (dry and wet) conditions of use was performed on the grafted samples. A set of thin films of type (A) and type (B) polymers spin-coated onto quartz slides were kept at room temperature and under daylight for over 2 months. Their habitus and absorbance/emission spectroscopic pattern remains unaltered. Another set of thin films of type (A) and type (B) polymers spin-coated onto quartz slides was immersed in aqueous solutions buffered at slightly acidic/basic pH (respectively at 5.0, 5.5, 6.5, 7.5, 8.0 pH values) for 2 weeks. As a result, the films do not detach from the quartz holder, and their habitus and spectroscopic absorbance/emission pattern remains unaltered. Finally, the ^1^H-NMR spectrum of all grafted samples recorded before and after 2 weeks resulted unaltered. In addition to quarts, various commercially available polymers were used as coating substrates.

**Figure 4:**
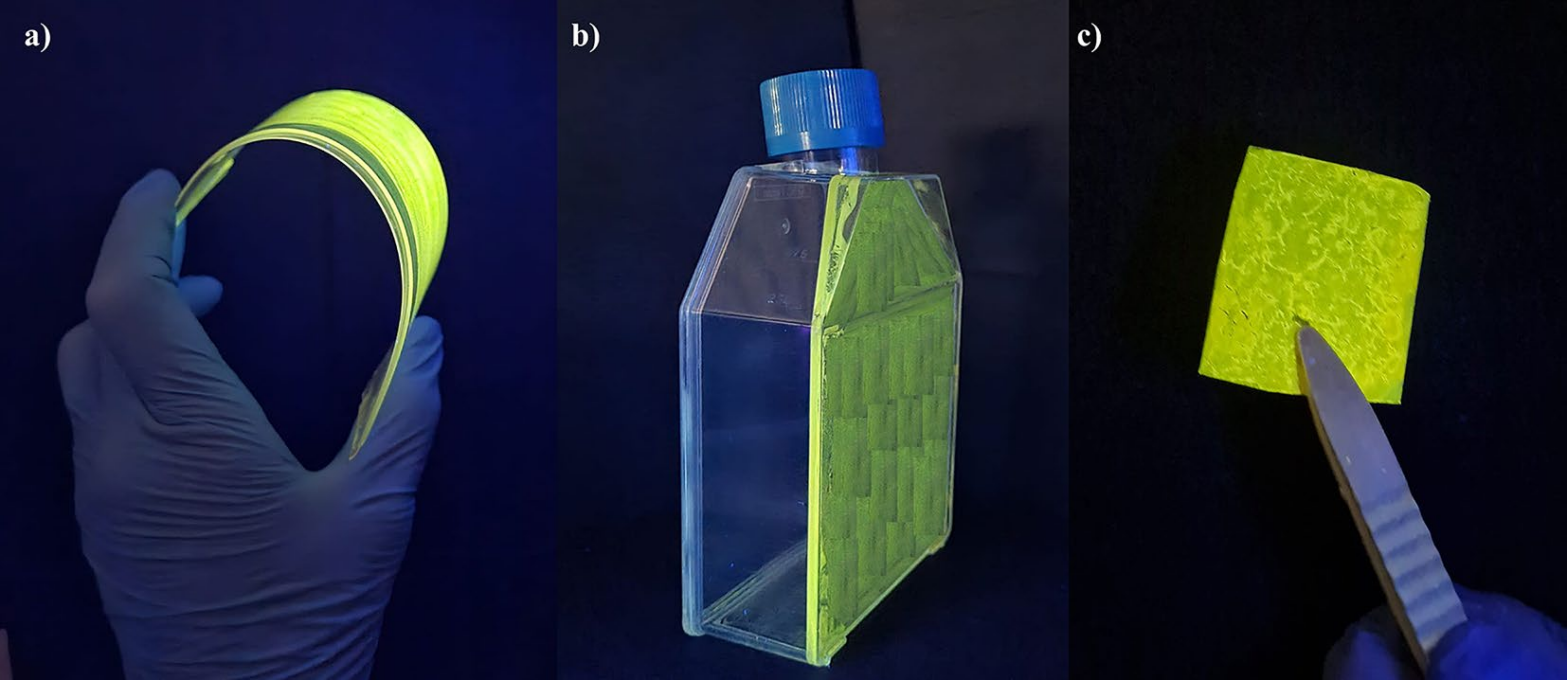
P1-Pd(A) deposed on a commercial PE flexible slide (**a**), on a BD falcon flask in PS (**b**), and turned into a die-cast film (**c**), irradiated by a UV–Visible lamp at 365 nm.

Interestingly, the films of the cyclopalladated polymers do not detach from commercial samples of PE, PMMA, PET, PVC, PS, PP and their habitus and spectroscopic pattern remain unaltered for months. As a practical check, P1-Pd(A) was coated onto a flexible commercial PE slide and on one face of a commercial laboratory flask (BD falcon for cell culture) made of PS. The deposition of a thin layer was trivially made by brushing a polymer solution in 1,1,2,2-TCE, as the solubility of P1-Pd(A) in 1,1,2,2-TCE at 50–60 °C is still sufficient for brushing the solution onto a support. In both cases, the emission can be well appreciated using a UV–Visible lamp at 365 nm (see Fig. 4).

Solubility is a relevant parameter for the polymers’ processability and coating operations. In our case, all polymers are insoluble in low-boiling organic solvents. Therefore, the hybrid polymeric layers are stable in contact with common solvents such as acetone, chloroform, ethyl acetate, and hexane. On the other hand, the organic polymers and the (A) type polymers can be dissolved in boiling 1,1,2,2-TCE or ortho dichlorobenzene (o-DCB), and their concerted solutions can be managed at 60 °C for the brushing/coating time. Contrarily, in the (B) type polymers, solubility drops, and the polymers cannot be easily processed. Finally, all the polymers can be extruded in fibres or die cast in plates by heating the solid material above T_g_ (200 °C). For example, in Fig. 4c is shown a small die-cast plate of P1-Pd(A) obtained from the solid sample using a heating press at 190 °C for 5 min. In all cases, the casting process can be carried out without damage.

**Figure 5:**
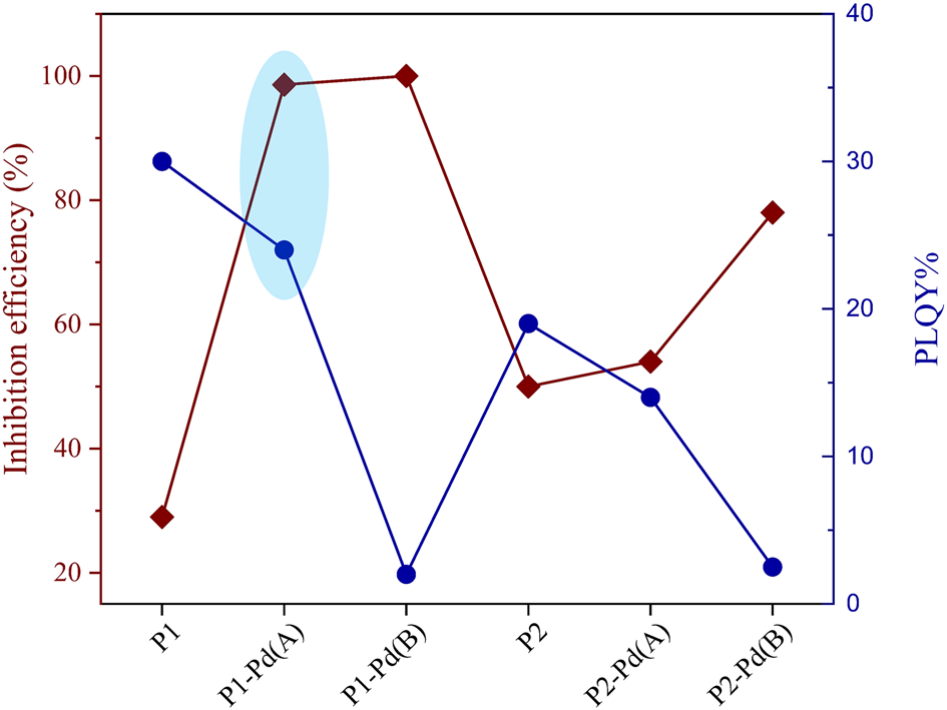
Summary of the main features of the polymers. The blue points represent the PL quantum yield values measured on polymer thin films (from Table 3). The red points represent the antibacterial inhibition efficiency against total coliform bacteria (from Table 1).

The diagram in Fig. 5 summarizes the main characteristics of the polymers in terms of monitorability (i.e., emission intensity quantified by PLQY value) and bacterial inhibition efficiency. The better performance (evidenced in the blue oval) is noticeable of P1-Pd(A) polymer which exhibits high values of both PLQY and inhibition efficiency.

### Theoretical analysis

**Figure 6:**
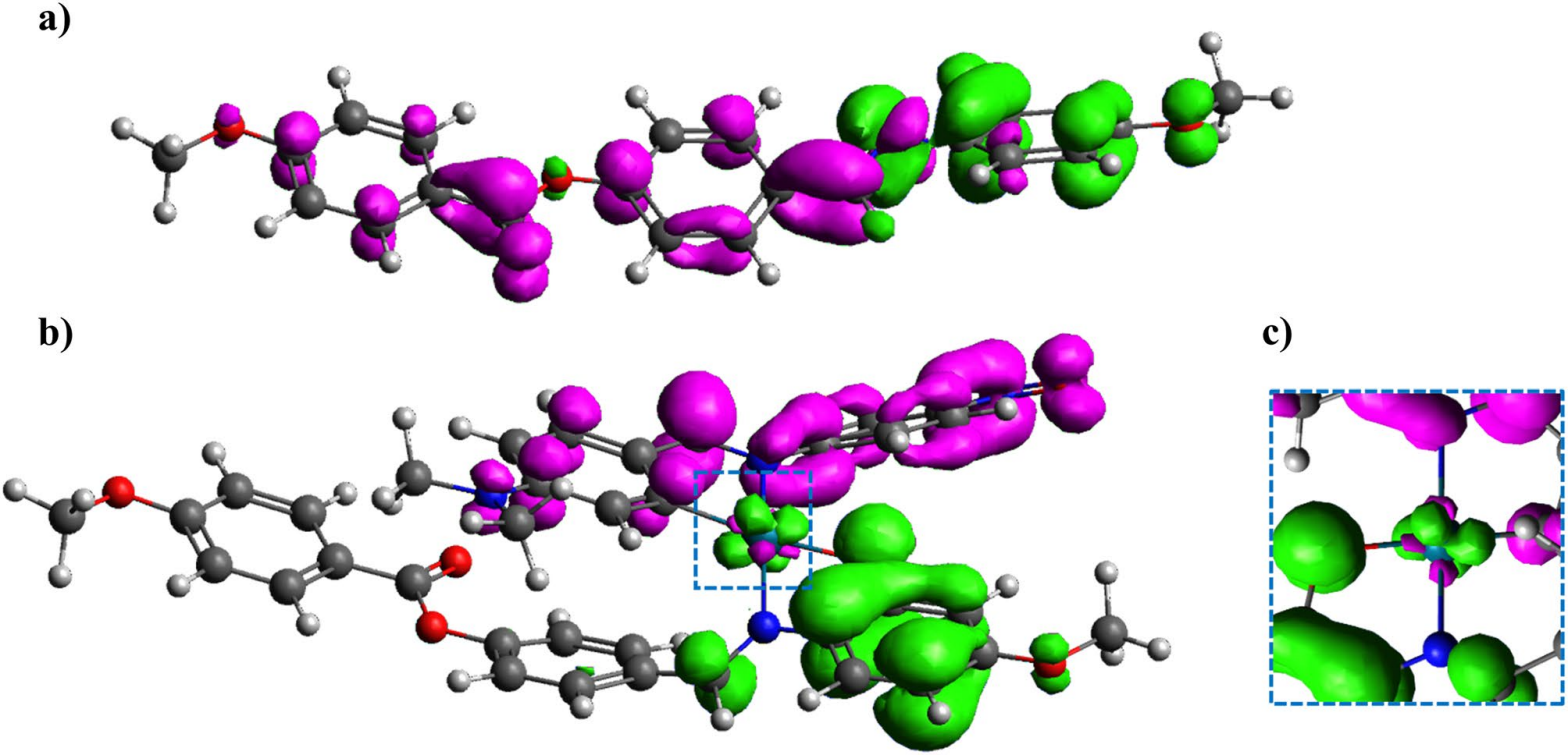
The isosurface plot is set to 0.002 |e|/a_0_ iso-level density. The magenta lobes are the positive contribution to the differential electron density r_diff_; the green lobes are the negative isovalues. In (**a**) is represented the P precursor, in (**b**) is represented P-Pd complex moiety. The enlarged detail of the differential electron density on the metal cation is in the inset (**c**).

The theoretical models, P and P-Pd, are simplified moieties resembling the aromatic part of the polymer (representative of both P1 and P2) ending with methyl groups, respectively, before (P) and after (P-Pd) coordination of the orthopalladated fragment (see Fig. 6). By the classical molecular orbital representation of the HOMO to LUMO transition, it could be hard to describe “which electrons” go from “which occupied orbitals” to “which unoccupied orbitals”. To overcome the issue, the excitation process was examined by the electronic charge transfer between the first excited state and the ground state using a more intuitive representation. The process was evaluated considering the differential electron density. The differential electron contributions (r_diff_ = r_exc_ − r_gr_) is the electron transfers from the first excited state and the ground state. The electron differences between HOMO and LUMO are represented as the density plot in Fig. 6, where are clearly detectable the charge transfer process and the involved molecular sites.

A significant difference is detected for P compared to P-Pd. This is because the main electron charge transfer contribution in the HOMO–LUMO excitation of the precursor P entails a redistribution of the electronic path involving the oxygen of the chain as the charge donor (negative differential densities represented as the green lobes in Fig. 6a) and the aromatic groups (positive differential densities represented as the magenta lobes in Fig. 6a).

In the case of P-Pd (Fig. 6b), the excitation process involves the charge transfer from the organometallic moiety Pd-L to the *O, N* chelating core of P. A role can be ascribed to Pd (II) cation as an electronic bridge between the organic chelating site of P and the orthopalladated ligand L. The frontiers orbitals localized on the metal cation are shown in the inset, Fig. 6c.

Finally, the energy gap (eV) in the HOMO–LUMO transition of P compared to P-Pd underwent a significant decrease from 3.64 eV of P precursor to 2.03 eV of P-Pd complex moiety.

## Conclusions

In the search for new bio-responsive tools, hybrid materials obtained by the integration of a coordination fragment into an organic matrix play a role. By grafting an orthopalladated fragment to the *O*, *N* chelating site of two Schiff base polymers, we obtained two series of organic-metallorganic polymers. The Pd-L moiety was added in two different percentages with the aim of preserving the AIE activity and improving the bacterial inhibition ability of the organic polymers. While the most concentrated grafted samples suffer a sudden decline of PLQYs, the most diluted grafted samples still retain medium PLQYs. All hybrid samples display good-to-excellent activity against the total coliform group of urban wastewater, increased as compared to the related organic polymers. The best balance between PL performance and antimicrobial activity was obtained with the P1-Pd(A) sample, which shows almost total bacterial abatement and retains 24% PLQY in the solid state. These results suggest such a hybrid set as a stable, monitorable, and antimicrobial polymer for functional coatings.

### Supplementary Information

Supplementary Figures.

## Data Availability

The data presented in this study are available on request from the corresponding author.

## References

[1] Aldalbahi A, Periyasami G, Alrehaili A (2021). Synthesis of high molar extinction coefficient push-pull tricyanofuran-based disperse dyes: Biological activity and dyeing performance. New J. Chem..

[2] Khan MN, Parmar DK, Das D (2021). Recent applications of azo dyes: A paradigm shift from medicinal chemistry to biomedical sciences. Mini-Rev. Med. Chem..

[3] Shi Y, Yang Z, Xing L, Zhang X, Li X, Zhang D (2021). Recent advances in the biodegradation of azo dyes. World J. Microbiol. Biotechnol..

[4] Würthner F, Kaiser TE, Saha-Möller CR (2011). J-aggregates: From serendipitous discovery to supramolecular engineering of functional dye materials. Angew. Chem. Int. Ed..

[5] Periyan D, Chinnasamy U (2021). Maleimide-g-pyridine octadecene polymers: Synthesis, characterization, thermal stability, fluorescence and biological studies. Asian J. Chem..

[6] Diana R, Panunzi B, Shikler R, Nabha S, Caruso U (2019). A symmetrical azo-based fluorophore and the derived salen multipurpose framework for emissive layers. Inorg. Chem. Commun..

[7] Zhang J, Xue F, Wang Z (2022). AIE-active fluorescent porous polymers for recognizable detection of imidacloprid and structure-property relationship. Chem. Mater..

[8] Saha B, Ruidas B, Mete S, Mukhopadhyay CD, Bauri K, De P (2020). AIE-active non-conjugated poly(N-vinylcaprolactam) as a fluorescent thermometer for intracellular temperature imaging. Chem. Sci..

[9] Yu Y, Xing H, Zhou Z, Liu J, Sung HHY, Williams ID, Halpert JE, Zhao Z, Tang BZ (2022). How do molecular interactions affect fluorescence behavior of AIEgens in solution and aggregate states?. Sci. China Chem..

[10] Alam P, Leung NLC, Zhang J, Kwok RTK, Lam JWY, Tang BZ (2021). AIE-based luminescence probes for metal ion detection. Coord. Chem. Rev..

[11] Diana R, Panunzi B (2021). Zinc (Ii) and aiegens: The “clip approach” for a novel fluorophore family. A review. Molecules.

[12] Ghosh R, Jayakannan M (2023). Theranostic FRET gate to visualize and quantify bacterial membrane breaching. Biomacromolecules.

[13] Hu R, Yang X, Qin A, Tang BZ (2021). AIE polymers in sensing, imaging and theranostic applications. Mater. Chem. Front..

[14] Wang Z, Geng H, Nie C, Xing C (2022). Conjugated polymers for combatting antimicrobial resistance. Chin. J. Chem..

[15] Haktaniyan M, Bradley M (2022). Polymers showing intrinsic antimicrobial activity. Chem. Soc. Rev..

[16] Jain A, Duvvuri LS, Farah S, Beyth N, Domb AJ, Khan W (2014). Antimicrobial polymers. Adv. Healthcare Mater..

[17] Singh A, Tiwari A, Bajpai J, Bajpai AK, Singh A, Tiwari A, Bajpai J, Bajpai AK (2017). Polymer-based antimicrobial coatings as potential biomaterials: From action to application. Handbook of Antimicrobial Coatings.

[18] Serrano-Aroca Á, Cano-Vicent A, Sabater i Serra R, El-Tanani M, Aljabali AA, Tambuwala MM, Mishra YK (2022). Scaffolds in the microbial resistant era: Fabrication, materials, properties and tissue engineering applications. Mater. Today Biol..

[19] Wang CG, Surat'man NEB, Mah JJQ, Qu C, Li Z (2022). Surface antimicrobial functionalization with polymers: Fabrication, mechanisms and applications. J. Mater. Chem. B.

[20] Yuan H, Li Z, Wang X, Qi R (2022). Photodynamic antimicrobial therapy based on conjugated polymers. Polymers.

[21] Pham P, Oliver S, Boyer C (2023). Design of antimicrobial polymers. Macromol. Chem. Phys..

[22] Lackner M, Guggenbichler JP, Lackner M, Guggenbichler JP (2013). Antimicrobial surfaces. Ullmann's Encyclopedia of Industrial Chemistry.

[23] Bobbarala V (2012). A Search for Antibacterial Agents.

[24] Videira-Quintela D, Martin O, Montalvo G (2021). Recent advances in polymer-metallic composites for food packaging applications. Trends Food Sci. Technol..

[25] Sun C, Wang X, Dai J, Ju Y (2022). Metal and metal oxide nanomaterials for fighting planktonic bacteria and biofilms: A review emphasizing on mechanistic aspects. Int. J. Mol. Sci..

[26] Panda S, Hajra S, Kaushik A, Rubahn HG, Mishra YK, Kim HJ (2022). Smart nanomaterials as the foundation of a combination approach for efficient cancer theranostics. Mater. Today Chem..

[27] Uddin MN, Ahmed SS, Alam SMR (2020). Review: Biomedical applications of Schiff base metal complexes. J. Coord. Chem..

[28] Gourdon L, Cariou K, Gasser G (2022). Phototherapeutic anticancer strategies with first-row transition metal complexes: A critical review. Chem. Soc. Rev..

[29] Paprocka R, Wiese-Szadkowska M, Janciauskiene S, Kosmalski T, Kulik M, Helmin-Basa A (2022). Latest developments in metal complexes as anticancer agents. Coord. Chem. Rev..

[30] Concilio S, Ferrentino I, Sessa L, Massa A, Iannelli P, Diana R, Panunzi B, Rella A, Piotto S (2017). A novel fluorescent solvatochromic probe for lipid bilayers. Supramol. Chem..

[31] Pervaiz M, Munir A, Riaz A, Saeed Z, Younas U, Imran M, Ullah S, Bashir R, Rashid A, Adnan A (2022). Review article—Amalgamation, scrutinizing, and biological evaluation of the antimicrobial aptitude of thiosemicarbazide Schiff bases derivatives metal complexes. Inorg. Chem. Commun..

[32] Diana R, Panunzi B, Tuzi A, Caruso U (2019). Two tridentate pyridinyl-hydrazone zinc(II) complexes as fluorophores for blue emitting layers. J. Mol. Struct..

[33] Ceramella J, Iacopetta D, Catalano A, Cirillo F, Lappano R, Sinicropi MS (2022). A review on the antimicrobial activity of schiff bases: Data collection and recent studies. Antibiotics.

[34] Matsumoto Y, Sawamura J, Murata Y, Nishikata T, Yazaki R, Ohshima T (2020). Amino acid schiff base bearing benzophenone imine as a platform for highly congested unnatural α-amino acid synthesis. J. Am. Chem. Soc..

[35] Lowe CR, Burton SJ, Pearson JC, Clonis YD, Stead V (1986). Design and application of bio-mimetic dyes in biotechnology. J. Chromatogr. B Biomed. Sci. Appl..

[36] Stasiak N, Kukula-Koch W, Glowniak K (2014). Modern industrial and pharmacological applications of indigo dye and its derivatives—A review. Acta Pol. Pharm..

[37] Waziri I, Yusuf TL, Akintemi E, Kelani MT, Muller A (2023). Spectroscopic, crystal structure, antimicrobial and antioxidant evaluations of new Schiff base compounds: An experimental and theoretical study. J. Mol. Struct..

[38] Aldorkee SY, Abdul A, Al-Janabi HS (2022). Evaluation of Aminoacetophenoneoxime derivatives of oxime Schiff bases as a new antimicrobial agent. World J. Microbiol. Biotechnol..

[39] Shi J, Song F, Ge H, Gao Y, Guo S (2022). Synthesis, characterization and antimicrobial property in vitro of supramolecular coordination polymers bearing brominated Schiff base ligand. J. Inorg. Biochem..

[40] Matsumoto T, Kamino M, Yamada R, Konishi Y, Ogino H (2020). Identification of genes responsible for reducing palladium ion in *Escherichia coli*. J. Biotechnol..

[41] Dechouk LF, Bouchoucha A, Abdi Y, Si Larbi K, Bouzaheur A, Terrachet-Bouaziz S (2022). Coordination of new palladium (II) complexes with derived furopyran-3,4-dione ligands: Synthesis, characterization, redox behaviour, DFT, antimicrobial activity, molecular docking and ADMET studies. J. Mol. Struct..

[42] Fernández-Arias M, Vilas AM, Boutinguiza M, Rodríguez D, Arias-González F, Pou-Álvarez P, Riveiro A, Gil J, Pou J (2022). Palladium nanoparticles synthesized by laser ablation in liquids for antimicrobial applications. Nanomaterials.

[43] Chen Y, Chen Z, Yang D, Zhu L, Liang Z, Pang Y, Zhou L (2022). Novel microbial palladium nanoparticles with a high photothermal effect for antibacterial applications. ACS Omega.

[44] Mostafaii G, Chimehi E, Gilasi H, Iranshahi L (2017). Investigation of zinc oxide nanoparticles effects on removal of total coliform bacteria in activated sludge process effluent of municipal wastewater. J. Environ. Sci. Technol..

[45] Caruso U, Panunzi B, Roviello A, Tingoli M, Tuzi A (2011). Two aminobenzothiazole derivatives for Pd(II) and Zn(II) coordination: Synthesis, characterization and solid state fluorescence. Inorg. Chem. Commun..

[46] Caruso U, Diana R, Panunzi B, Roviello A, Tingoli M, Tuzi A (2009). Facile synthesis of new Pd(II) and Cu(II) based metallomesogens from ligands containing thiophene rings. Inorg. Chem. Commun..

[47] Cariati F, Caruso U, Centore R, De Maria A, Fusco M, Panunzi B, Roviello A, Tuzi A (2004). New NLO active cyclopalladated chromophores in main-chain polymers. Inorg. Chim. Acta.

[48] Diana R, Gentile FS, Carella A, Di Costanzo L, Panunzi B (2022). Insights into two novel orthopalladated chromophores with antimicrobial activity against *Escherichia coli*. Molecules.

[49] Diana R, Gentile FS, Concilio S, Petrella A, Belvedere R, Schibeci M, Arciello A, Di Costanzo L, Panunzi B (2023). A DR/NIR hybrid polymeric tool for functional bio-coatings: Theoretical study, cytotoxicity, and antimicrobial activity. Polymers.

[50] Caruso U, Panunzi B, Roviello A, Sirigu A (1994). Networks from liquid crystalline segmented chain polymers. Macromolecules.

[51] Neese F (2012). The ORCA program system. Wiley Interdiscipl. Rev. Comput. Mol. Sci..

[52] Raghavachari K (2000). Perspective on "Density functional thermochemistry. III. The role of exact exchange". Theor. Chem. Acc..

[53] Diana R, Caruso U, Di Costanzo L, Gentile FS, Panunzi B (2021). Colorimetric recognition of multiple first-row transition metals: A single water-soluble chemosensor in acidic and basic conditions. Dyes Pigm..

[54] Gentile FS, Salustro S, Desmarais JK, Ferrari AM, D'Arco P, Dovesi R (2018). Vibrational spectroscopy of hydrogens in diamond: A quantum mechanical treatment. Phys. Chem. Chem. Phys..

[55] Weigend F (2006). Accurate Coulomb-fitting basis sets for H to Rn. Phys. Chem. Chem. Phys..

[56] Salustro S, Gentile FS, Erba A, Carbonniére P, El-Kelany KE, Dovesi R (2018). The characterization of the VNxHy defects in diamond through the infrared vibrational spectrum. A quantum mechanical investigation. Carbon.

[57] Diana R, Caruso U, Di Costanzo L, Bakayoko G, Panunzi B (2020). A novel DR/NIR T-shaped aiegen: Synthesis and x-ray crystal structure study. Crystals.

[58] Wang H, Zhang L, Jin X, Tian P, Ding X, Chang J (2022). A water-soluble fluorescent probe for monitoring mitochondrial GSH fluctuations during oxidative stress. RSC Adv..

[59] Cui WL, Wang MH, Yang YH, Qu J, Zhang H, Zhu X, Wang JY (2022). A water-soluble polymer fluorescent probe via RAFT polymerization for dynamic monitoring of cellular lipid droplet levels and zebrafish imaging. New J. Chem..

[60] Chowdhury P, Banerjee A, Saha B, Bauri K, De P (2022). Stimuli-responsive aggregation-induced emission (aie)-active polymers for biomedical applications. ACS Biomater. Sci. Eng..

[61] Wang W, Mattoussi H (2020). Engineering the bio-nano interface using a multifunctional coordinating polymer coating. Acc. Chem. Res..

[62] Tapia-Hernández JA, Torres-Chávez PI, Ramírez-Wong B, Rascón-Chu A, Plascencia-Jatomea M, Barreras-Urbina CG, Rangel-Vázquez NA, Rodríguez-Félix F (2015). Micro- and nanoparticles by electrospray: Advances and applications in foods. J. Agric. Food Chem..

[63] Bazhina ES, Bovkunova AA, Shmelev MA, Korlyukov AA, Pavlov AA, Hochvaldová L, Kvítek L, Panáček A, Kopel P, Eremenko IL, Kiskin MA (2023). Zinc(II) and copper(II) complexes with N-substituted imines derived from 4-amino-1,2,4-triazole: Synthesis, crystal structure, and biological activity. Inorg. Chim. Acta.

[64] Palaniammal A, Vedanayaki S (2022). Synthesis, spectral characterization, biological activity of macrocyclic ligands and metal complexes derived from 3,4-diaminobenzophenone and diketones. Rasayan J. Chem..

[65] El-Razek SEA, El-Gamasy SM, Hassan M, Abdel-Aziz MS, Nasr SM (2020). Transition metal complexes of a multidentate Schiff base ligand containing guanidine moiety: Synthesis, characterization, anti-cancer effect, and anti-microbial activity. J. Mol. Struct..

[66] Yan Y, Zhang J, Ren L, Tang C (2016). Metal-containing and related polymers for biomedical applications. Chem. Soc. Rev..

[67] Ulla F, Yashoda M (2018). Synthesis and spectral studies on substituted metal (II)-tetra-1-(thiophene-2-yl) methaniminephthalocyanine complexes. Orient. J. Chem..

[68] Borbone F, Caruso U, Causà M, Fusco S, Panunzi B, Roviello A, Shikler R, Tuzi A (2014). Series of O, N, O-tridentate ligands zinc(II) complexes with high solid-state photoluminescence quantum yield. Eur. J. Inorg. Chem..

[69] Caruso U, Panunzi B, Roviello A, Tuzi A (2013). Fluorescent metallopolymers with Zn(II) in a Schiff base/phenoxide coordination environment. Inorg. Chem. Commun..

[70] Kunkely H, Vogler A (2003). Excited state properties of dimeric π-allylpalladium(II) chloride. Photoreduction of palladium induced by ligand-to-metal charge transfer excitation. Inorg. Chim. Acta.

[71] Roviello A, Borbone F, Carella A, Diana R, Roviello G, Panunzi B, Ambrosio A, Maddalena P (2009). High quantum yield photoluminescence of new polyamides containing oligo-PPV amino derivatives and related oligomers. J. Polym. Sci. A.

[72] Manouras T, Koufakis E, Vasilaki E, Peraki I, Vamvakaki M (2021). Antimicrobial hybrid coatings combining enhanced biocidal activity under visible-light irradiation with stimuli-renewable properties. ACS Appl. Mater. Interfaces..

[73] Lu C, Wen T, Zheng M, Liu D, Quan G, Pan X, Wu C (2020). Poly(ethylene glycol) crosslinked multi-armed poly(l-lysine) with encapsulating capacity and antimicrobial activity for the potential treatment of infection-involved multifactorial diseases. Pharmaceutics.

[74] Inayat S, Ahmad SR, Awan SJ, Nawshad M, Ali Q (2023). In vivo and in vitro toxicity profile of tetrabutylammonium bromide and alcohol-based deep eutectic solvents. Sci. Rep..

[75] Flores-Rojas GG, López-Saucedo F, López-Barriguete JE, Isoshima T, Luna-Straffon M, Bucio E (2018). Polypropylene films modified by grafting-from of ethylene glycol dimethacrylate/glycidyl methacrylate using γ-rays and antimicrobial biofunctionalization by Schiff bases. MRS Commun..

[76] Saldarriaga Fernández IC, Mei HCVD, Metzger S, Grainger DW, Engelsman AF, Nejadnik MR, Busscher HJ (2010). In vitro and in vivo comparisons of staphylococcal biofilm formation on a cross-linked poly(ethylene glycol)-based polymer coating. Acta Biomater..

[77] Evelyn ZP, Neftalí RV, Isaac C, Luis Gilberto T (2013). Coliforms and helminth eggs removals by coagulation-flocculation treatment based on natural polymers. J. Water Resour. Prot..

[78] Mishra BK, Hati S, Das S, Prajapati JB (2019). Biofunctional attributes and storage study of soy milk fermented by *Lactobacillus rhamnosus* and *Lactobacillus helveticus*. Food Technol. Biotechnol..

[79] Kim I, Jin SM, Han EH, Ko E, Ahn M, Bang WY, Bang JK, Lee E (2017). Structure-dependent antimicrobial theranostic functions of self-assembled short peptide nanoagents. Biomacromolecules.

[80] Xie YY, Zhang YW, Qin XT, Liu LP, Wahid F, Zhong C, Jia SR (2020). Structure-dependent antibacterial activity of amino acid-based supramolecular hydrogels. Colloids Surf. B Biointerfaces.

[81] Zhang Y, Wei J, Qiu Y, Niu C, Song Z, Yuan Y, Yue T (2019). Structure-dependent inhibition of *Stenotrophomonas maltophilia* by polyphenol and its impact on cell membrane. Front. Microbiol..

[82] Shi Y, Chen T, Shaw P, Wang PY (2022). Manipulating bacterial biofilms using materiobiology and synthetic biology approaches. Front. Microbiol..

[83] Scharf S, Notz S, Jeschke J, Preuß A, Rüffer T, Wiese A, Künzel-Tenner A, Schulze S, Hietschold M, Lang H (2023). Bis(phosphine) Pd(II) and Pt(II) ethylene glycol carboxylates: Synthesis, nanoparticle formation, catalysis. Polyhedron.

[84] Liu J, Li F, Lu J, Li R, Wang Y, Wang Y, Zhang X, Fan C, Zhang R (2021). Atomically dispersed palladium-ethylene glycol-bismuth oxybromide for photocatalytic nitrogen fixation: Insight of molecular bridge mechanism. J. Colloid Interface Sci..

[85] Leung ACW, MacLachlan MJ (2007). Schiff base complexes in macromolecules. J. Inorg. Organomet. Polym Mater..

[86] Sukwattanasinitt M, Nantalaksakul A, Potisatityuenyong A, Tuntulani T, Chailapakul O, Praphairakait N (2003). An electrochemical sensor from a soluble polymeric Ni-salen complex. Chem. Mater..

[87] Liu X, Shang Y, Chen ZR (2021). Vinyl groups containing tetraphenylethylene derivatives as fluorescent probes specific for palladium and the quenching mechanism. Chin. J. Chem..

[88] Panchompoo J, Aldous L, Baker M, Wallace MI, Compton RG (2012). One-step synthesis of fluorescein modified nano-carbon for Pd(ii) detection via fluorescence quenching. Analyst.

[89] Panunzi B, Diana R, Concilio S, Sessa L, Shikler R, Nabha S, Tuzi A, Caruso U, Piotto S (2018). Solid-state highly efficient dr mono and poly-dicyano-phenylenevinylene fluorophores. Molecules.

[90] Borbone F, Caruso U, Palma SD, Fusco S, Nabha S, Panunzi B, Shikler R (2015). High solid state photoluminescence quantum yields and effective color tuning in polyvinylpyridine based zinc(II) metallopolymers. Macromol. Chem. Phys..

