# Supplementary material for: AIEgen orthopalladated hybrid polymers for efficient inactivation of the total coliforms in urban wastewater
Source: Sci Rep. 2023 Sep 22;13:15790. doi: 10.1038/s41598-023-41315-x (PMC10516893; doi:10.1038/s41598-023-41315-x)
Supplement: Supplementary file 1 — Supplementary Figures. [file 41598_2023_41315_MOESM1_ESM.pdf]

Supplementary Materials

AIEgen orthopalladated hybrid polymers for efficient inactivation of the total coliforms in urban wastewater

Lucia Sessa[1], Rosita Diana[2*], Francesco Silvio Gentile[3], Fabio Mazzaglia[4], and Barbara Panunzi[2]

[1] *Department of Pharmacy, University of Salerno, Via Giovanni Paolo II, 132, Fisciano, 84084, SA, Italy*

[2] *Department of Agricultural Sciences, University of Naples Federico II, Portici, NA, Italy*

[3] *Department of Chemical Sciences, University of Napoli Federico II, Strada Comunale Cinthia, 26, Napoli, 80126, Italy*

[4] *C.R.A. s.r.l., Calle Giovanni Legrenzi, 2 – 30171, VE, Italy*

***** Correspondence: rosita.diana@unina.it

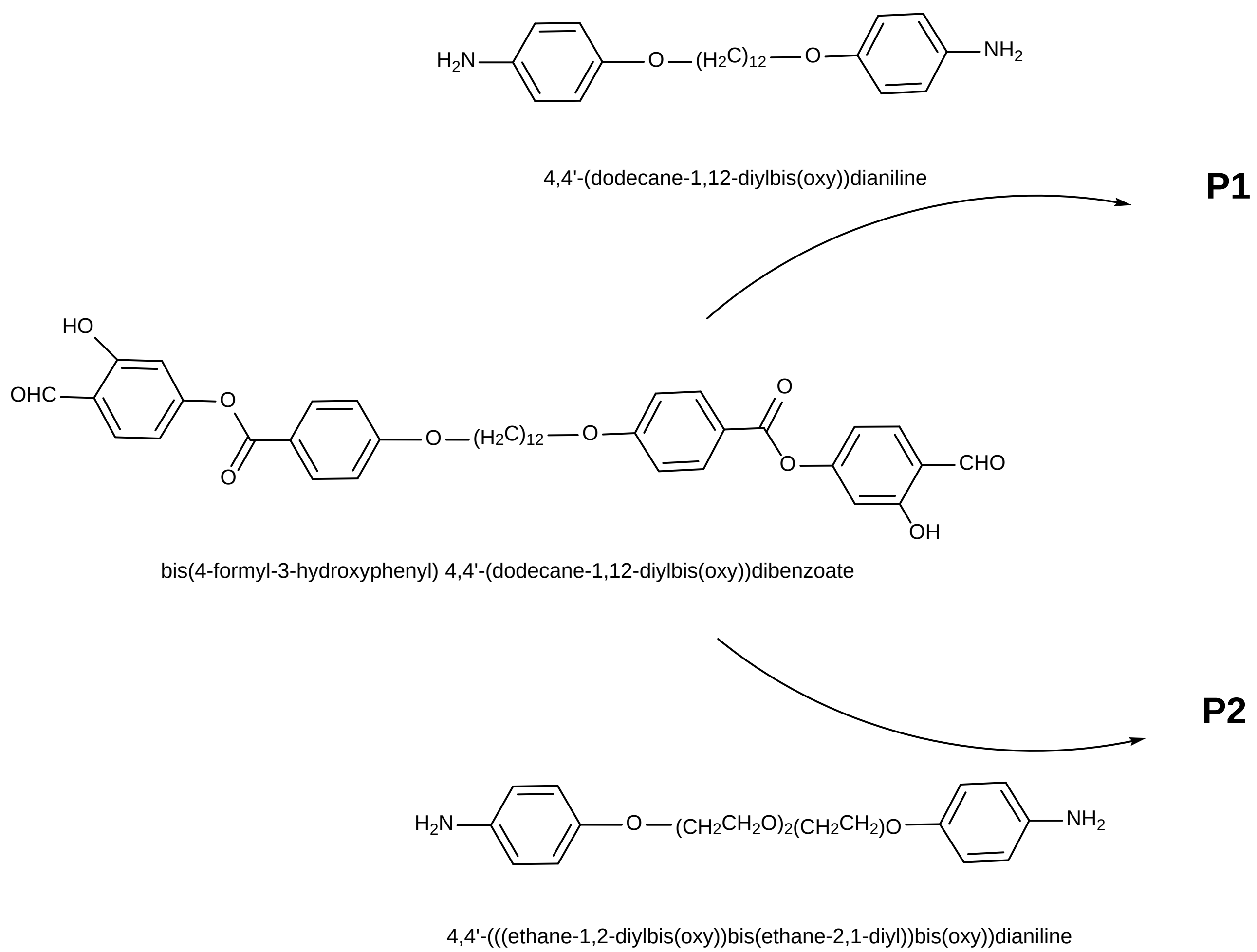

Figure S1. Synthetic scheme to polymers P1 and P2 starting from dialdehyde and dianiline monomers

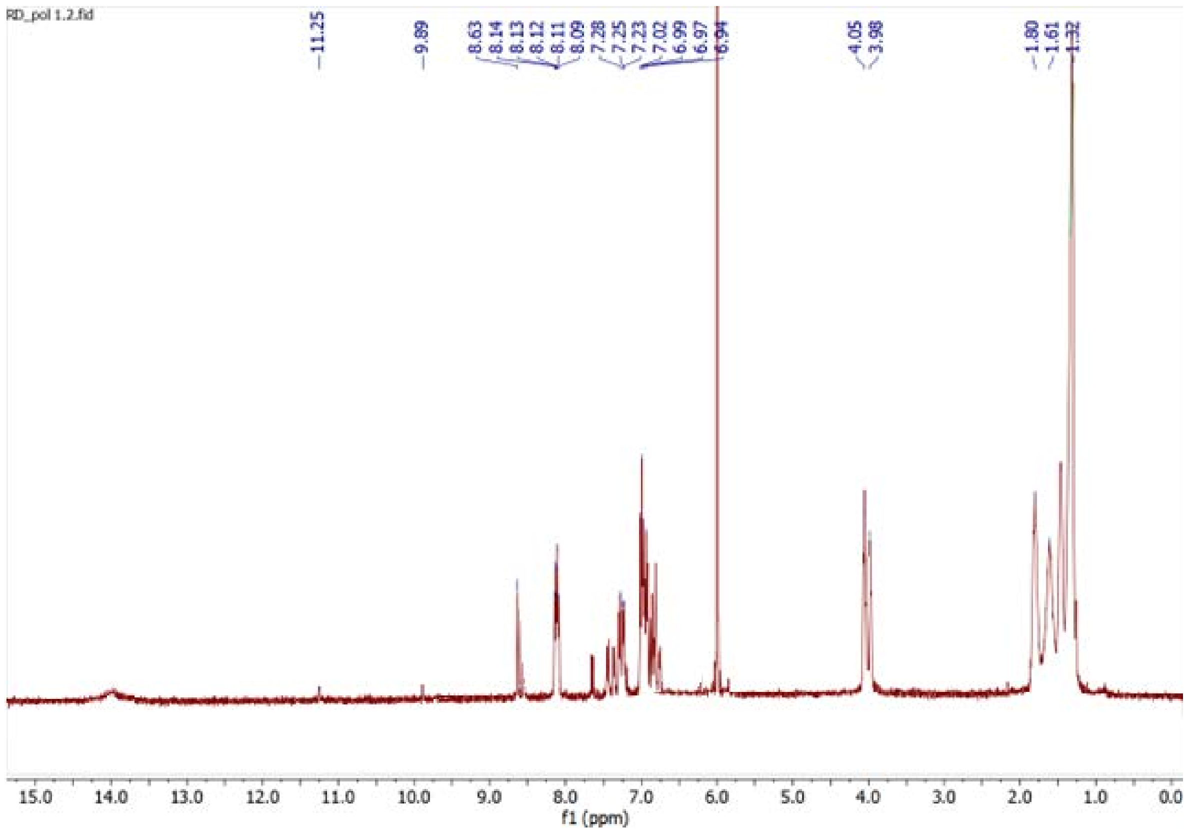

Figure S2. ^{1}H-NMR of P1 in TCE

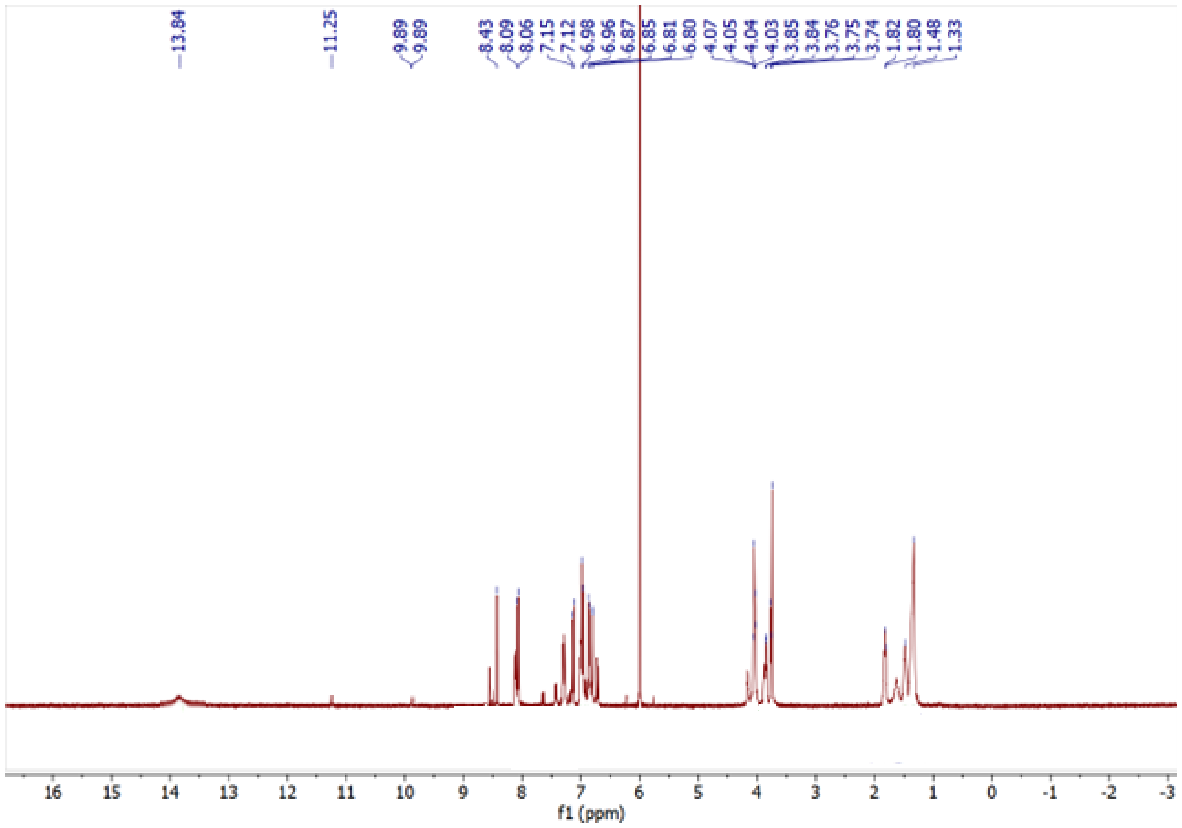

Figure S3 ^{1}H-NMR of P2 in TCE

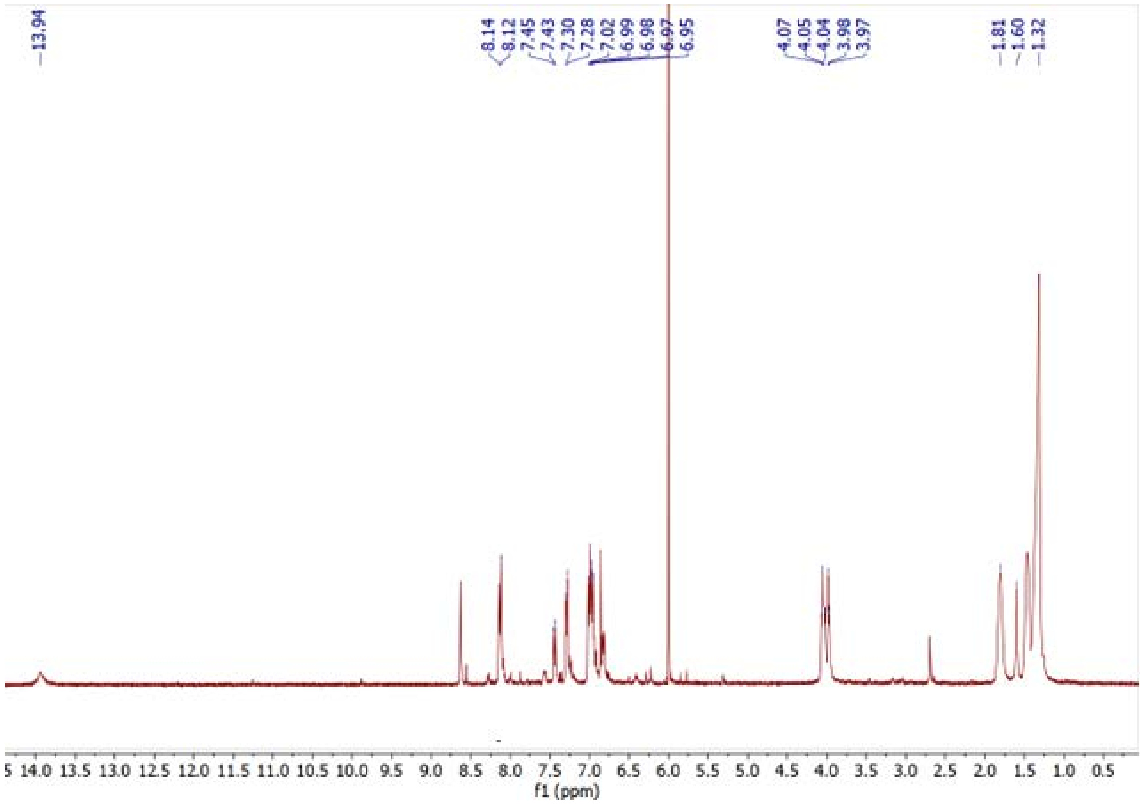

Figure S4. ^{1}H-NMR of P1-Pd(A) in TCE

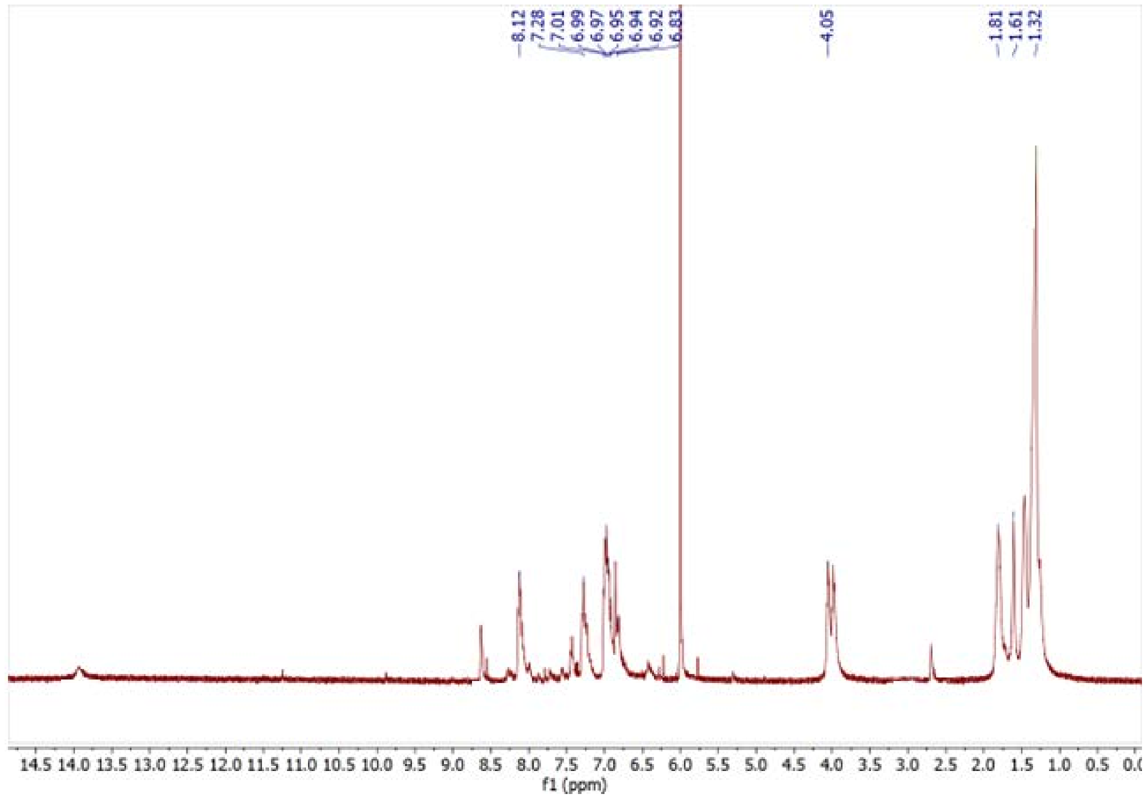

Figure S5. ^{1}H-NMR of P1-Pd(B) in TCE

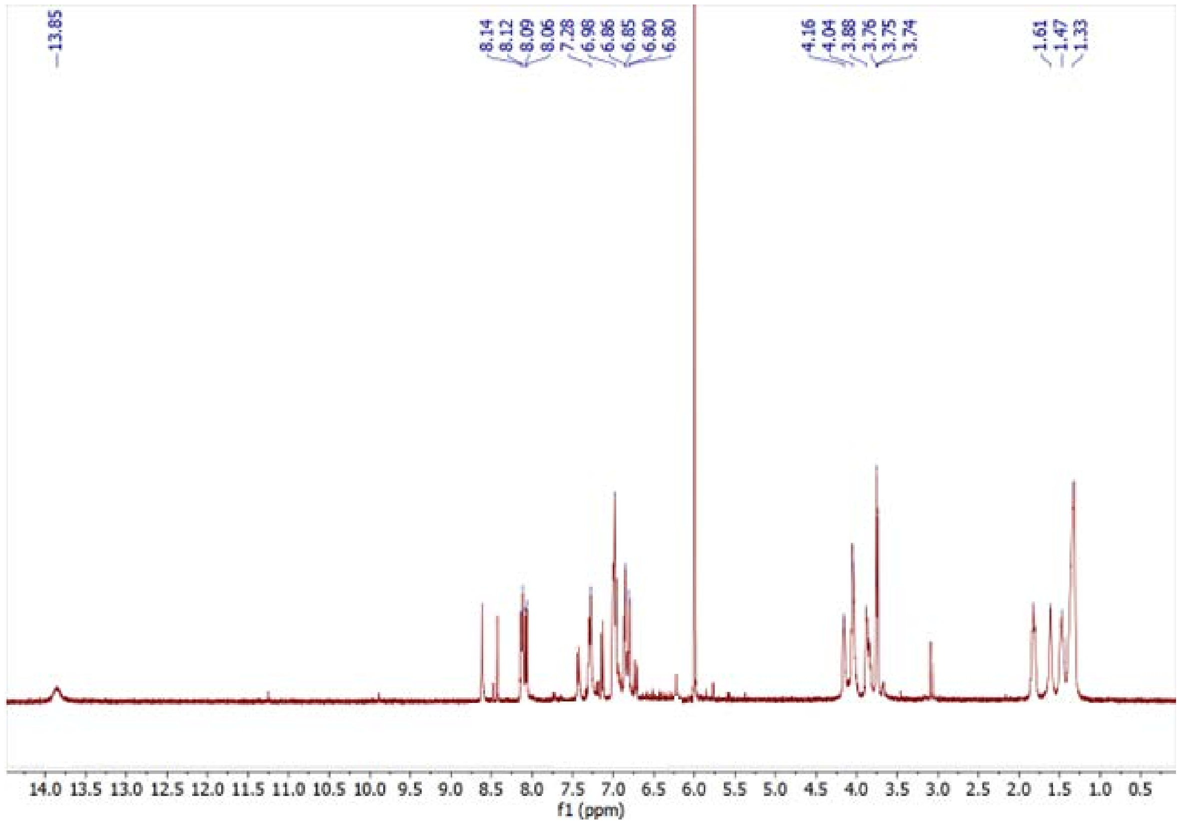

Figure S6. ^{1}H-NMR of P2-Pd(A) in TCE

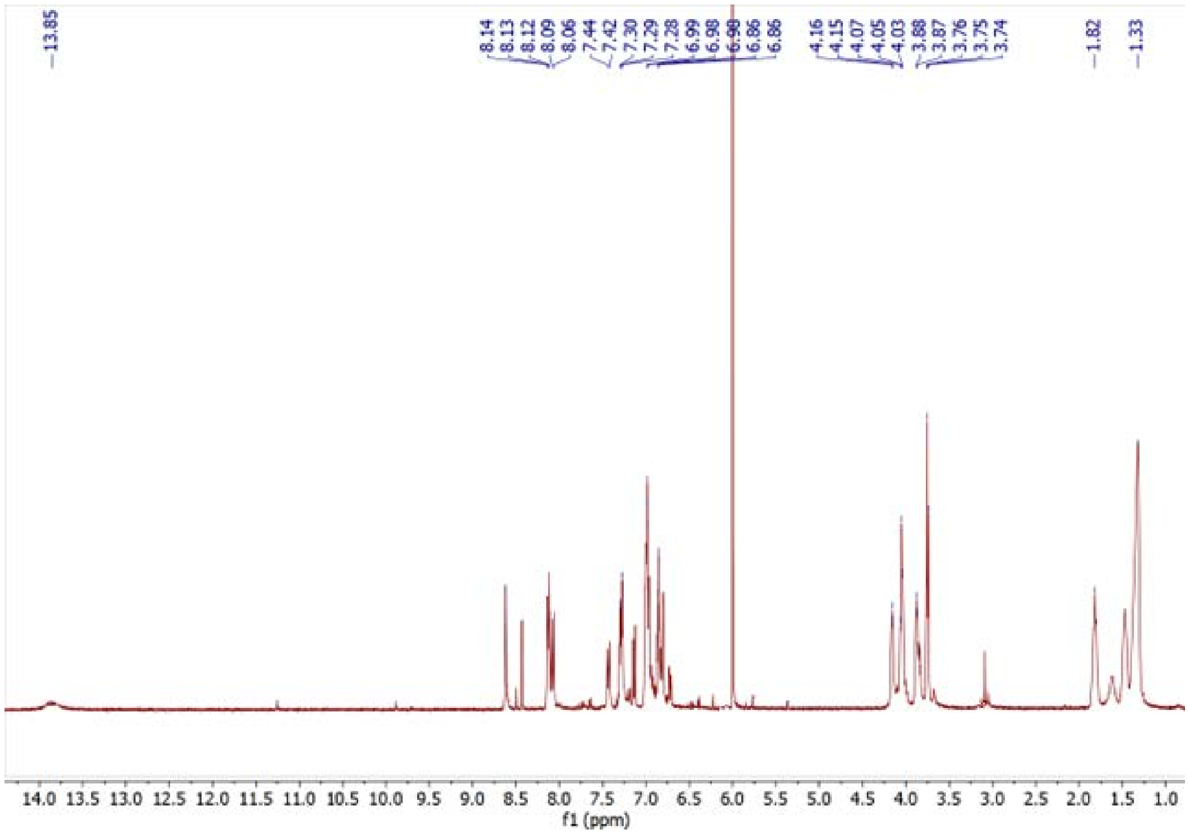

Figure S7. ^{1}H-NMR of P2-Pd(B) in TCE

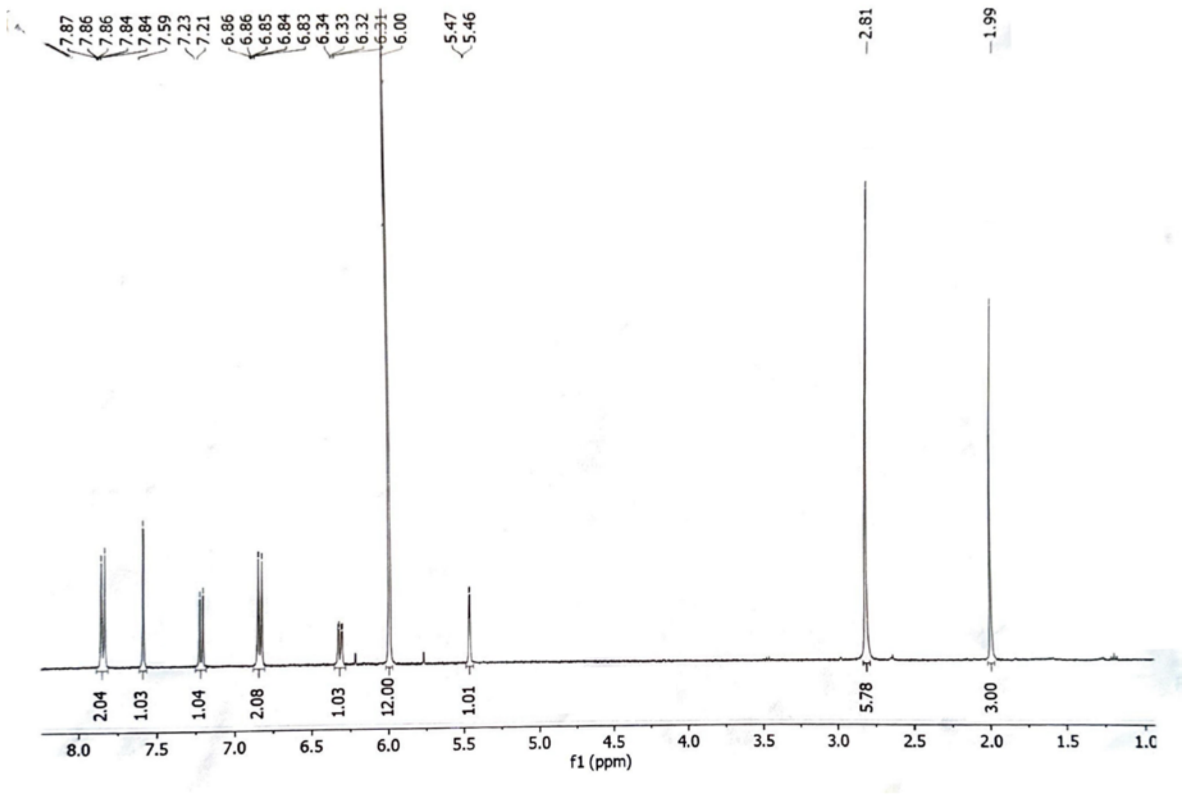

Figure S8. ^{1}H-NMR of D in TCE

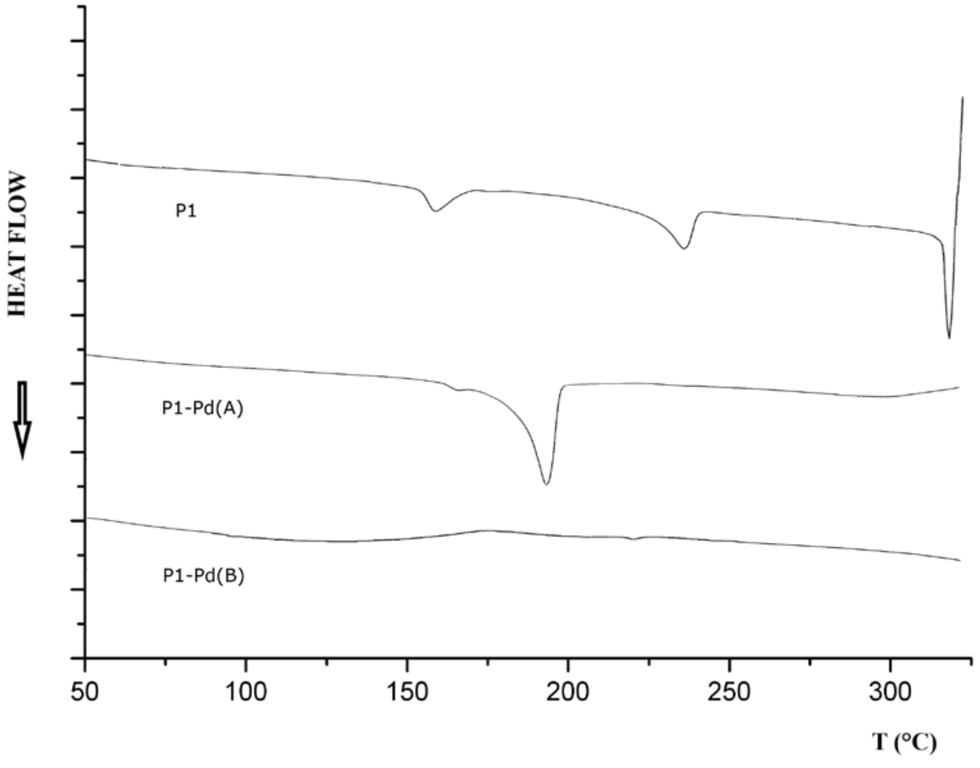

Figure S9. DSC curves of P1 and related grafted polymers

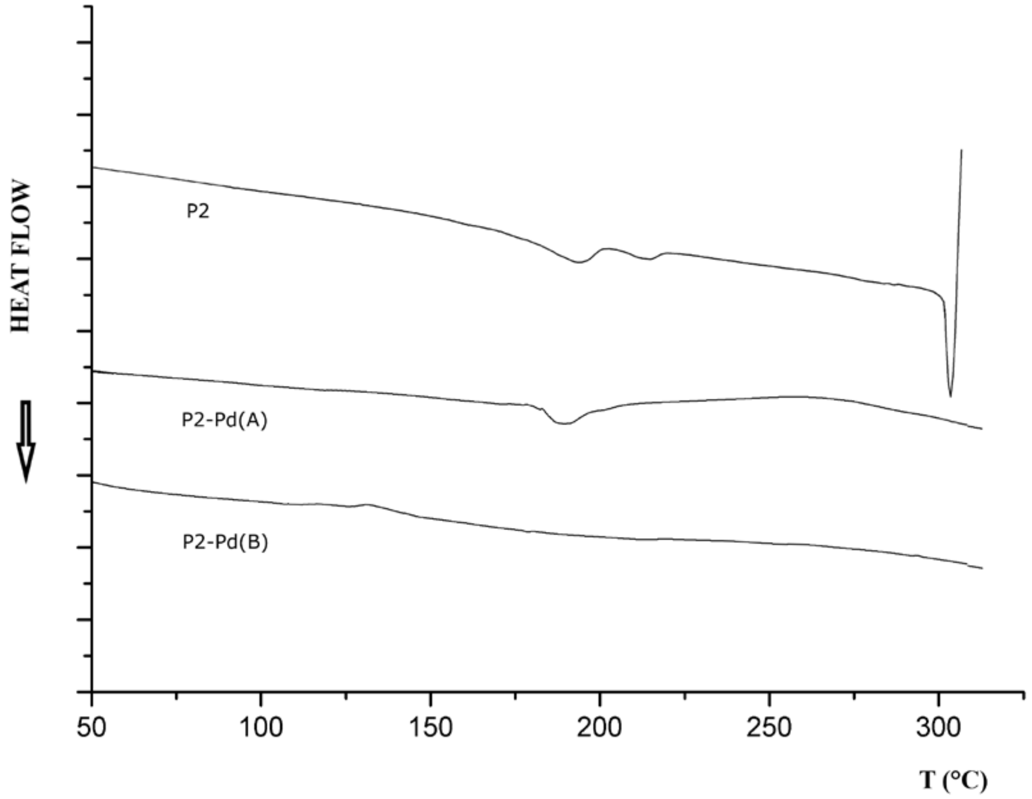

Figure S10. DSC curves of P2 and related grafted polymers

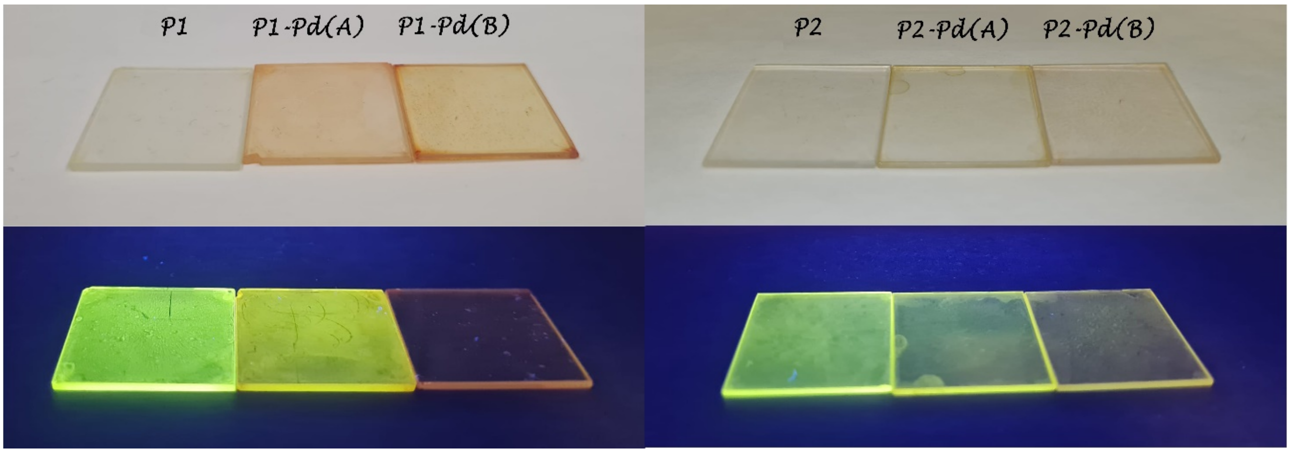

Figure S11. Polymeric thin films in natural light (above) and irradiated by a commercial UV-visible lamp at 365 nm (below).

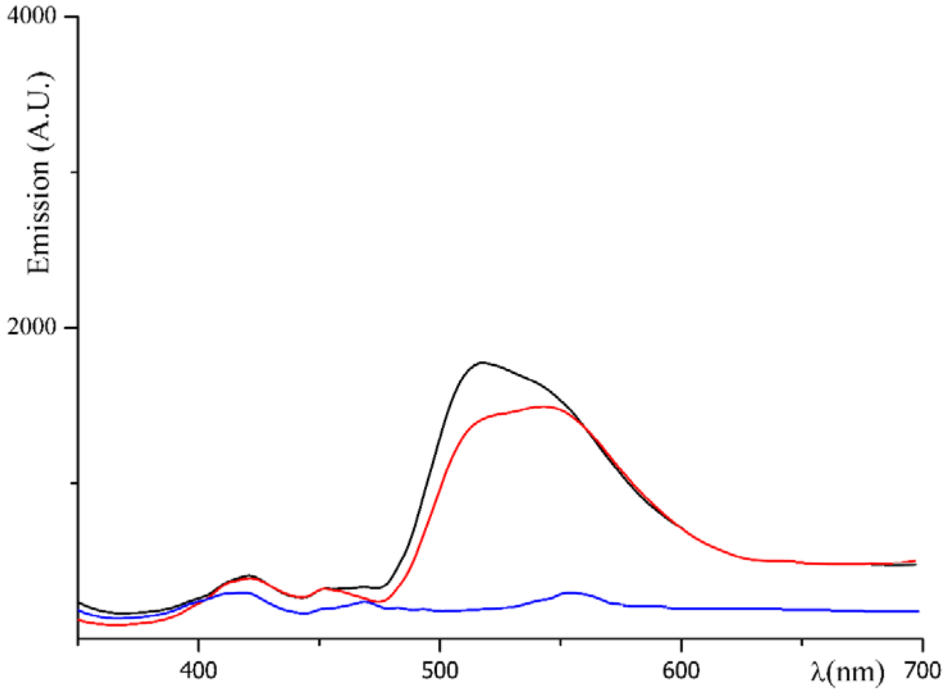

Figure S12. Emission spectra recorded on thin film of P1 (black curve), P1-Pd(A) (red curve), and P1-Pd(B) (blue curve).

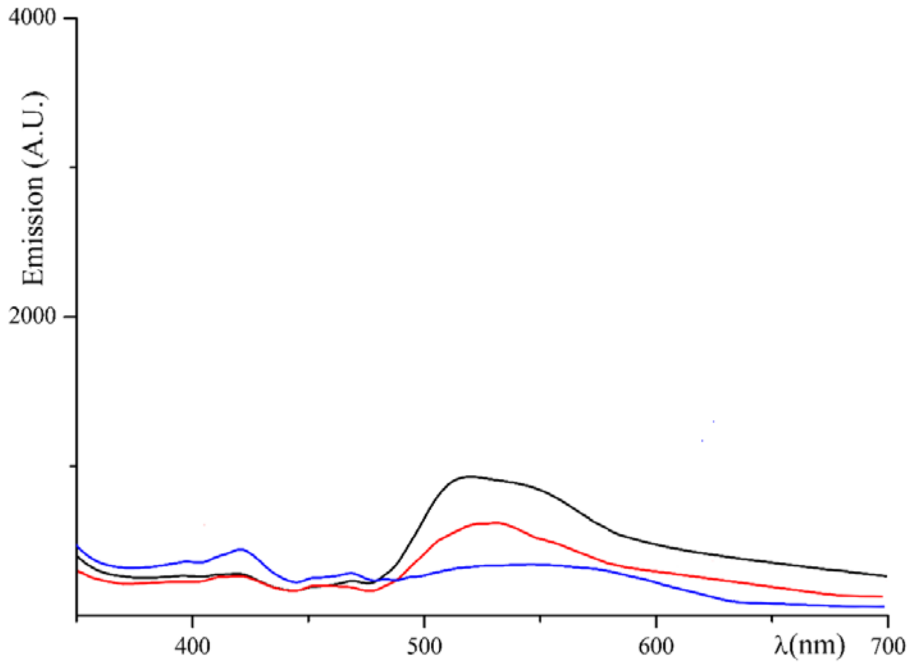

Figure S13. Emission spectra recorded on thin film of P2 (black curve), P2-Pd(A) (red curve), and P2-Pd(B) (blue curve).
